# Imatinib attenuates reperfusion injury in a rat model of acute myocardial infarction

**DOI:** 10.1007/s00395-022-00974-z

**Published:** 2023-01-13

**Authors:** Lara S. F. Konijnenberg, Tom T. J. Luiken, Andor Veltien, Laween Uthman, Carolien T. A. Kuster, Laura Rodwell, Guus A. de Waard, Mariska Kea-te Lindert, Anat Akiva, Dick H. J. Thijssen, Robin Nijveldt, Niels van Royen

**Affiliations:** 1 Department of Cardiology, Radboud University Medical Center, Geert Grooteplein 10, 6525 GA Nijmegen, The Netherlands; 2 Department of Physiology, Radboud Institute for Health Sciences, Radboud University Medical Center, Nijmegen, The Netherlands; 3 Department of Medical Imaging, Radboud University Medical Center, Nijmegen, The Netherlands; 4 Department of Epidemiology and Biostatistics, Radboud University Medical Center, Nijmegen, The Netherlands; 5 Department of Cardiology, Amsterdam University Medical Center, Amsterdam, The Netherlands; 6 Department of Cell Biology, Radboud Institute of Molecular Life Sciences, Radboud University Medical Center, Nijmegen, The Netherlands; 7 Electron Microscopy Center, Radboudumc Technology Center Microscopy, Radboud Institute of Molecular Life Sciences, Radboud University Medical Center, Nijmegen, The Netherlands; 8 Department of Biochemistry, Radboud Institute of Molecular Life Sciences, Radboud University Medical Center, Nijmegen, The Netherlands

**Keywords:** Acute myocardial infarction, Reperfusion injury, Microvascular injury, Langendorff, Cardiac magnetic resonance imaging

## Abstract

Following an acute myocardial infarction, reperfusion of an occluded coronary artery is often accompanied by microvascular injury, leading to worse long-term prognosis. Experimental studies have revealed the potential of tyrosine-kinase inhibitor imatinib to reduce vascular leakage in various organs. Here, we examined the potential of imatinib to attenuate microvascular injury in a rat model of myocardial reperfusion injury. Isolated male Wistar rat hearts (*n* = 20) in a Langendorff system and male Wistar rats (*n* = 37) in an in vivo model were randomly assigned to imatinib or placebo and subjected to ischaemia and reperfusion. Evans-blue/Thioflavin-S/TTC staining and Cardiac Magnetic Resonance Imaging were performed to assess the extent of reperfusion injury. Subsequently, in vivo hearts were perfused ex vivo with a vascular leakage tracer and fluorescence and electron microscopy were performed. In isolated rat hearts, imatinib reduced global infarct size, improved end-diastolic pressure, and improved rate pressure product recovery compared to placebo. In vivo, imatinib reduced no-reflow and infarct size with no difference between imatinib and placebo for global cardiac function. In addition, imatinib showed lower vascular resistance, higher coronary flow, and less microvascular leakage in the affected myocardium. At the ultrastructural level, imatinib showed higher preserved microvascular integrity compared to placebo. We provide evidence that low-dose imatinib can reduce microvascular injury and accompanying myocardial infarct size in a rat model of acute myocardial infarction. These data warrant future work to examine the potential of imatinib to reduce reperfusion injury in patients with acute myocardial infarction.

**Supplementary Information:**

The online version contains supplementary material available at 10.1007/s00395-022-00974-z.

## Introduction

ST-elevation myocardial infarction (STEMI) is a life-threatening condition that requires timely reperfusion to salvage the myocardium at risk. However, despite the high rate of succes of reperfusion therapy by primary percutaneous coronary intervention (PCI) [55], reperfusion itself can induce further cellular damage to the myocardium. This is known as ischaemia–reperfusion (IR) injury, a complex pathophysiological process involving many forms of damage (mechanical, biochemical or inflammatory) to different types of cardiac cells, impeding restoration of myocardial perfusion [21, 22, 32].

In patients, IR injury can be best visualised by Cardiac Magnetic Resonance Imaging (CMR) [5]. In almost half of the successful revascularised STEMI patients, CMR shows a typical pattern of a contrast-void infarct core in the contrast-enhanced infarct area on late gadolinium enhancement imaging [61]. This specific form of IR injury involves massive microvascular injury (MVI), distal microembolisation, inflammation and haemorrhage [6, 23, 29, 32, 50], which is associated with an increased risk of the development of heart failure and cardiovascular death [10, 61]. This paradigm has shifted emphasis towards the coronary microcirculation as a promising target in cardioprotection, and underlines the importance of the development of new therapeutic approaches to limit MVI [19, 21, 32].

One of the hallmarks of MVI is the loss of vascular integrity, which becomes already apparent in the first hours of reperfusion [23]. At the ultrastructural level, MVI manifests as loss of endothelial cell–cell junctions, thinning and rupture of endothelial cells, extravasation of erythrocytes in the surrounding interstitial space [23], and, when left untreated, intramyocardial haemorrhage (IMH) [50]. IMH worsens cardiac function and induces pro-inflammatory factors [41], which further intensifies IR injury. Since IMH develops over time during reperfusion and even drives final infarct size [36], it is key to intervene at an early timepoint during reperfusion to limit the development of severe MVI and IMH. Despite the increasing knowledge about the substantial impact of MVI, studies directly investigating the coronary microvasculature as culprit in IR injury are limited [17, 34].

Here we investigated the role of imatinib in STEMI-related MVI and vascular leakage. It has been reported that imatinib inhibits multiple tyrosine kinases of which the Abelson-related gene (Arg/Abl2) is proposed as the most important target affecting vascular leakage [2, 3, 9, 49]. Notably, pre-clinical studies found that imatinib attenuated vascular leakage in mouse models treated with permeability-inducing factors in skin, lung and sepsis [3], preserved peripheral microcirculatory perfusion in rats with cardiopulmonary bypass [33], and attenuated oedema formation in models of pulmonary IR injury [37, 57]. Interestingly, besides a reduction in perivascular oedema, imatinib was able to limit reperfusion-induced pulmonary haemorrhage and neutrophil invasion in rats [57]. These findings suggest that imatinib may also protect the coronary microcirculation, thereby preventing severe MVI and IMH, and subsequent myocardial damage.

In this study, we investigate the effects of imatinib on the coronary microvasculature, myocardial infarct size and global cardiac function in an ex vivo and in vivo rat model of acute myocardial infarction. We hypothesise that treatment with imatinib attenuates MVI and reduces infarct size.

## Methods

### Animals

This study was performed under project licence (AVD10300 2016 774) authorised by the Central Authority for Scientific Procedures on Animals in the Netherlands. All experimental procedures were approved by the Animal Welfare Body of Radboudumc and performed following the guidelines from Directive 2010/63/EU of the European Parliament. Male Wistar rats (RccHan:WIST, Envigo, Horst, the Netherlands), 9–13 weeks old were used for the experiments. They were housed in individually ventilated cages with standard cage enrichment in groups of a maximum of four, in a temperature-controlled room (20–24 ℃; 45–65% humidity) under 12/12 h light/dark cycle and provided with food and water ad libitum.

The software programme Castor Electronic Data Capture (Castor EDC, v2022.4.1.3, Amsterdam, the Netherlands) was used for registration, randomisation, blinding and data storage.

### Ischaemia–reperfusion ex vivo

Rats (*n* = 24) were randomly assigned to imatinib (10 µM; #SML1027, Sigma-Aldrich) or placebo (0.9% NaCl). Concentration of imatinib was chosen based on previous dose–response experiments regarding endothelial barrier function [3]. At the start of the experiment, the rats were treated with 20 IU heparin (Heparin Leo 5000 IU/mL) intraperitoneally and 0.02 mg/kg buprenorphine (Bupaq 0.3 mg/mL) subcutaneously. The rats were intubated and mechanically ventilated (PhysioSuite™, Kent Scientific) with 3% isoflurane (pressure-controlled, positive end inspiratory pressure 12–15 cmH_2_O, positive end expiratory pressure 2 cmH_2_O, fraction of inspired O_2_ 20%, 70–80 strokes/minute, observed tidal volume 5–15 mL/kg bodyweight). Anaesthetic depth was assessed by multiple toe pinches. Isolation of the hearts and ex vivo perfusion in the Langendorff system was performed as described previously [60]. In short, the hearts were isolated by in-situ aortic cannulation, connected to the Langendorff system and perfused with oxygenated (95% O_2_ and 5% CO_2_), 37 ℃ heated modified Krebs–Henseleit buffer (118 mM NaCl, 4.7 mM KCl, 1.2 mM MgSO_4_, 1.25 mM CaCl_2_, 1.2 mM KH_2_PO_4_, 25 mM NaHCO_3_, 11 mM glucose, 0.5 mM ethylenediaminetetraacetic acid (EDTA)). A small incision in the left atrium was made to insert the left ventricular balloon to assess left ventricular function. Diastolic pressure was adjusted to 5–10 mmHg. Perfusion pressure was set at 80 mmHg. The hearts were placed in a heated organ bath containing Krebs–Henseleit buffer. No external cardiac pacing was performed. After 20 min of stabilisation and 15 min of baseline perfusion, the hearts were subjected to 40 min global ischaemia, followed by 120 min reperfusion. The hearts were infused with imatinib or placebo for 15 min during baseline and the first 5 min of reperfusion. Left ventricular function was assessed by rate pressure product recovery and end-diastolic pressure at the end of reperfusion. Exclusion criteria were based on previously published guidelines for pre-clinical studies on cardioprotection [7] and included a heart isolation period of four minutes or longer, a baseline coronary flow of less than 10 mL/min, rupture of left ventricular balloon during the experiment, and ventricular fibrillation during baseline recordings.

### Infarct staining

Immediately after the experiment, the apex of each heart was snap-frozen in liquid nitrogen and stored for additional protein analysis. Subsequently, hearts were sliced in ~ 8–10 consecutive slices of 1 mm across the transverse axis. Slices were incubated with 2,3,5-triphenyl tetrazolium chloride staining (TTC, Sigma-Aldrich) at 37 ℃ and fixed in 4% formalin at room temperature (RT) overnight. The following day, the heart slices were scanned at 1200 dots per inch and global infarct size, as percentage of total mass, was analysed using Fiji software (1.51n) [52]. Investigators were blinded for treatment group.

### LDH activity assay

Coronary effluent was collected at *t* = 5, 10, 15, 30, 45, 60, 90 and 120 min reperfusion to determine lactate dehydrogenase (LDH) activity as a measure of cellular injury. LDH activity was determined by 400 µL coronary effluent, 2 mL LDH assay buffer (0.1 mM KH_2_PO_4_, 386.4 nM polyethylene glycol tert-octylphenyl ether (Triton X-100, Sigma-Aldrich), 135.7 µM nicotinamide adenine dinucleotide hydrogen (NADH, Roche Diagnostics GmbH) in 100 mL MilliQ, pH 7.5) and 25 µL pyruvate, and detected by a colorimetric assay (OD340). LDH activity was calculated from the slope of the curve of NADH to NAD^+^ conversion and corrected for heart wet weight and the corresponding coronary flow immediately before the sample was obtained. Investigators were blinded for treatment group.

### Western blot analysis

#### Protein determination

In brief, heart apex samples were weighed and lysis buffer (including 50 mM tris-hydrochloride pH 7.5, 1 mM egtazic acid, 1 mM EDTA, 1% *(v/v)* Triton X-100 ,  10 mM sodium glycerophosphate, 1 mM sodium orthovanadate, 50 mM sodium fluoride, 10 mM sodium pyrophosphate, 270 mM sucrose, 150 mM NaCl, pepstatin 1:1000, leupeptin 1:1000, phenylmethylsulfonylfluoride 1:100, and aprotinin 1:5000) was added to the samples. Samples were homogenised and lysates were centrifuged at 13,000 rounds per minute at 4 ℃ for 15 min. Protein concentration was measured using the Bradford Protein Assay (ThermoFisher Scientific) and determined with Bio-Rad. Finally, a mixture of Laemmli (containing 10% *(w/v)* sodium dodecyl sulphate, 50% *(v/v)* glycerol, 100 mM dithiothreitol, 0.05% *(w/v)* bromophenol blue, and 300 mM tris-hydrochloride), dithiothreitol, and MilliQ was added to the supernatant.

#### Western blot

Protein samples (10 µg in 10 µl) and Precision Plus Blue marker (Bio-Rad) were loaded on 4–15% mini-PROTEAN TGX Stain-Free Protein Gels (Bio-Rad) for electrophoresis and transferred to a polyvinylidene difluoride membrane using a Trans-blot Turbo Transfer System (Bio-Rad). Non-specific binding was blocked with 5% (*w*/*v*) non-fat dry milk (Elk) in a mixture of tris-buffered saline and polysorbate 20 (TBS-T) for 1 h at RT. The membranes were incubated with the primary antibodies in 1% (*w*/*v*) milk in TBS-T (Vascular Endothelial (VE)-cadherin #19–3600, 1:500, ThermoFisher Scientific and glyceraldehyde 3-phosphate dehydrogenase (GAPDH #AM4300, 1:5000, ThermoFisher Scientific) overnight at 4 °C. The membranes were washed four times with TBS-T, incubated with the secondary antibodies in 1% (*w*/*v*) milk in TBS-T (anti-rabbit IRdye 680 or anti-mouse IRdye 800, respectively, 1:1000) for 1 h at RT and washed 5 times with TBS-T. The membranes were scanned with Odyssey ClX (Li-cor). Finally, western blots were analysed using Fiji to calculate the relative intensity of each band, corrected for GAPDH.

### Non-infarcted control rats

Rats without an acute myocardial infarction (AMI) (*n* = 5) were included as CMR control group to define reference values. Rats were scanned at similar age with identical CMR scan protocol as rats with AMI induced by left anterior descending coronary artery (LAD) ligation.

### Ischaemia–reperfusion in vivo

Rats (*n* = 48) were randomly assigned to imatinib (30 mg/kg; #SML1027, Sigma-Aldrich) or placebo intravenously, blinded for the investigators. The imatinib dose was based on rat dose equivalency calculations of the clinically approved dose in humans [40]. Thirty minutes before surgery, the rats were treated with 0.02 mg/kg buprenorphine (Bupaq 0.3 mg/mL) subcutaneously. The rats were intubated and mechanically ventilated as described for the ex vivo experiments. Anaesthetic depth was assessed by multiple toe pinches. Rats were placed on a thermostatic heating pad and body temperature was continuously monitored (ThermoStar, RWD Life Sciences) using a rectal temperature probe. Rats were connected to various monitoring systems; Einthoven I electrocardiogram (ECG; FE136 Animal Bio Amp, ADInstruments), heart rate, tidal volume and oxygen saturation were monitored throughout the experiment. A tail vein cannula (BD Venflon PRO 22G, Becton Dickinson Infusion Therapy) was placed for infusion of imatinib or placebo and collection of blood samples. Xylocaine spray (AstraZeneca) was applied on the shaved skin and the operating area was disinfected using Betadine. A left thoracotomy was performed between the third and fourth rib and the pericardium was carefully opened to bring the LAD in sight. Imatinib or placebo (max. 1 mL, based on bodyweight) was injected intravenously via the tail vein cannula. The LAD was ligated ~ 1–2 mm below the left atrium with a 6–0 silk suture (Ethicon) 15–20 min after imatinib or placebo injection. Early indicators for successful ischaemia were the appearance of ST-elevation on the ECG recording and blanching of the heart distal from the ligation. After 45 min ischaemia, the ligature was released, air was removed from the thorax and the chest was closed. In vivo reperfusion was maintained for 180 min. Rats were included for final analysis if there were signs of ischaemia on ECG during the LAD ligation.

### Blood sampling and analysis

Blood samples were collected at baseline, end of ischaemia, and end of reperfusion (15, 60, and 240 min after imatinib/placebo injection, respectively). Samples were collected in Microvette^®^ EDTA-coated or lithium-heparin vials (Sarstedt), centrifuged for 15 min at 4600 rounds per minute and stored at − 80 ℃. Concentration of imatinib and N-desmethyl imatinib (479.6 g/mol, #16947–500, Sanbio), the main pharmacologically active metabolite of imatinib, were determined by liquid chromatography-tandem mass spectrometry as described previously [45]. Imatinib-d3 (496 g/mol, #18257–500, Sanbio) was used as internal standard. Blood samples at the end of reperfusion were used for the assessment of cardiac troponin-T and LDH. Concentration of troponin-T was determined with the high sensitive troponin-T STAT Reagent Kit (Roche Diagnostics GmbH) according to the manufacturer’s protocol. LDH was determined by spectrophotometry as described before.

### Cohort A: area at risk, infarct size, and no-reflow

In this series of experiments (*n* = 23), heparin (0.4 mL; 20U) was administered intravenously via the tail vein cannula at the end of 180 min reperfusion. Subsequently, the hearts were isolated by in-situ aortic cannulation and perfused with 37 ℃ heated modified Krebs–Henseleit buffer (118.5 mM NaCl, 4.7 mM KCl, 1.2 mM CaCl_2_, 25 mM NaHCO_3_, 1.2 mM MgCl_2_, 1.2 mM KH_2_PO_4_, 11 mM glucose). Filtered 4% Thioflavin-S solution (#T1892, Sigma-Aldrich) demarcated the area of no-reflow (Thioflavin-S negative area). The LAD was re-occluded and 2% Evans-blue solution (#E2129, Sigma-Aldrich) delineated the area at risk (Evans blue-negative area). The heart was rinsed with cold saline and sliced in 7 consecutive slices perpendicular to the ventricular long axis. Slices were photographed under ultraviolet light (λ = 366 nm) at fixed height, same magnification and including a scale grid. Finally, the hearts were incubated in TTC with dextran and photographed as described above to calculate the infarct area (TTC-negative area). Of each slice, specific areas were calculated and averaged for both sides of the slice: total area of left ventricle, area at risk, area of no-reflow, and infarct area. Area at risk was calculated as percentage of left ventricle, infarct size as percentage of area at risk, and area of no-reflow as percentage of infarct size, according to the guidelines for pre-clinical studies on cardioprotection [7]. Slices were manually analysed using Fiji (1.51n), blinded for treatment group.

### Cohort B: CMR imaging acquisition and analysis

In this series of experiments, rats (*n* = 25) were transported to a Bruker ClinScan 7 T horizontal bore MR system, interfaced to a Siemens Syngo VB15 console (Bruker BioSpin) equipped with a volume coil with an inner diameter of 114 mm and a surface coil with a diameter of 30 mm. Rats were placed in supine position and imaged two hours after the start of reperfusion.

Rats were mechanically ventilated (3% isoflurane, fraction of inspired O_2_ 30%) with a respiratory rate of 50 strokes/minute. Subsequently, rats were connected to a 3-lead ECG with respiration sensor. Core body temperature was maintained at 37 °C using a fibre-optical temperature probe and heated air. During cardiac imaging heart rate, respiratory rate and temperature were monitored using dedicated software (Small Animal Instruments Inc).

To study myocardial function, cine images were acquired with a 2D gradient-echo sequence (typical voxel size 0.313 × 0.313 × 1 mm^3^ and 14 phases per cardiac cycle) with prospective electrocardiographic gating. The heart was imaged in three planes: short axis (SAX), 2 chamber long axis (2CH) and 4 chamber long axis (4CH). Consecutive SAX images were acquired from base to apex to measure left ventricular (LV) volumes. To study the extent of oedema, a T2 map was calculated from a multi spin echo sequence with three different echo times (5, 10, 15 ms) in SAX at the mid-ventricular level. To study infarct size, an extracellular gadolinium-based contrast agent (Dotarem, Guerbet, Roissy, CdG, France, 0.4 mmol/kg) was injected via the tail vein cannula, two hours after the start of reperfusion. Late gadolinium enhancement (LGE) imaging was acquired using a 2D inversion recovery fast low angle shot sequence (typical voxel size 0.408 × 0.313 × 2 mm^3^) and performed 10 min after contrast injection. Consecutive SAX images were acquired from base to apex to measure infarct size, at identical slice positions as the cine SAX slices. All scanning parameters are shown in Supplemental Table 1.

### CMR analysis

Cine, LGE and T2 mapping analyses were performed offline using commercially available software (Medis, QMass 8.1 software, Medis Medical Imaging Systems, version 3.2.36.2) blinded for treatment group. Epicardial and endocardial contours were manually drawn in LV end-systolic and LV end-diastolic phase on the SAX images. Papillary muscles were excluded from the endocardium. Stroke volume (SV) was calculated as end-diastolic volume (EDV) minus end-systolic volume (ESV), left ventricular ejection fraction (LVEF) was calculated as SV divided by EDV, and cardiac output (CO) was calculated as SV multiplied by heart rate. Infarct area was defined as a mean signal intensity of at least five standard deviations above the mean signal intensity of remote myocardium and was manually corrected afterwards. Total infarct size was calculated as infarct area multiplied by slice thickness and expressed as a percentage of left ventricular mass (%LV). T2 values were recorded from the quantitative T2 maps using a region of interest in the infarct area and the remote area (i.e., the area 180° opposite to the infarct area).

### CMR feature tracking analysis

Cardiac strain is defined as the change in endocardial length between end-systolic and end-diastolic phase and is measured on the standard cine images (Medis, Qstrain software, Medis Medical Imaging Systems, version 3.2.36.2). Endocardial contours were manually drawn in the end-systolic and end-diastolic phase and contours were automatically tracked in consecutive frames. Left ventricular global longitudinal strain (GLS) was calculated as the average of strain measured on the 2CH and 4CH cines. Global circumferential strain was measured as the average strain on the basal-, mid- and apical SAX cines.

### Ex vivo coronary perfusion

After 180 min in vivo reperfusion, heparin (0.4 mL; 20U) was administered intravenously via the tail vein cannula. The Langendorff system enables ex vivo administration of fluorescent microspheres (0.1 µm, FluoSpheres™ carboxylate-modified, 540/560, ThermoFisher Scientific) or gold nanoparticles (0.1 µm, #742031, Sigma-Aldrich), which were used for the quantification of early vascular leakage. The hearts were isolated by in-situ aortic cannulation, connected to the Langendorff system and perfused with oxygenated (95% O_2_ and 5% CO_2_), 37 ℃ heated modified Krebs–Henseleit buffer (118.5 mM NaCl, 4.7 mM KCl, 1.2 mM CaCl_2_, 25 mM NaHCO_3_, 1.2 mM MgCl_2_, 1.2 mM KH_2_PO_4_, 11 mM glucose). Perfusion pressure was steadily increased to 80 mmHg. After 10 min of stabilisation, fluorescent microspheres (3.6 × 10^12^ in 1 mL) were slowly added to the perfusion medium. To remove microspheres from inside of the vasculature, perfusion with Krebs–Henseleit buffer was continued for 5 min*.* Mean coronary flow (mL/min) was calculated and corrected for perfusion pressure. To calculate vascular resistance (mmHg*min/mL), perfusion pressure (mmHg) was divided by mean coronary flow (mL/min).

### Tissue preparation for imaging

After ex vivo perfusion, the heart was removed from the cannula, submerged in ice-cold saline to induce cardioplegia, and sliced into five equal slices of 2 mm, starting from the apex as number 1 and the most basal slice as number 5. The mid-ventricular slices (e.g., slice 2,3,4) were either snap-frozen in optimal cutting temperature compound (Tissue-Tek OCT; Sakura Finetek) for immunohistochemistry or fixed in 2% glutaraldehyde in 0.1 M cacodylate buffer (pH 7.4) for electron microscopy.

### Immunohistochemistry

Of each heart, the mid-ventricular slices were used for immunohistochemistry. Frozen heart slices were sliced in 5 µm sections across the transversal axis. Sections were fixed in ice-cold acetone at − 20 ℃ for 10 min and rehydrated in phosphate buffered saline (PBS) for 15 min. Tissue was permeabilised with 0.1% *(v/v)* Triton X-100 and non-specific binding was blocked with 5% heat-inactivated normal donkey serum in 1% bovine serum albumin in PBS (PBSA) 1 h at RT. Endothelial cells were stained with Isolectin-B4 (10 µg/mL, #L2895, Sigma-Aldrich) and endothelial cell junctions with VE-cadherin (1:50, #36–1900, ThermoFisher Scientific) in 1% PBSA overnight at 4 ℃. Subsequently, slices were incubated with donkey anti-rabbit Alexa-647 (1:400, #A31573, ThermoFisher Scientific) and nuclei were stained with 4′,6-diamidino-2-phenylindole (DAPI) or Hoechst (1:5000, ThermoFisher Scientific) for 1 h at RT. Slices were mounted in Fluorescent Mounting Medium. The slides were scanned using confocal fluorescence microscope (LSM900, Zeiss microscopy GmbH) with the 5 × objective using the tile scan mode for overview images and the 20 × objective to assess detailed VE-cadherin expression.

### Immunofluorescence analysis

Determination of the infarct area was based on the infarct location on CMR LGE imaging. Percentage of area (%area) occupied with fluorescent microspheres and/or VE-cadherin was calculated in 3–5 regions of interest, with a fixed frame of 1*1 mm, in the infarct area (Supplemental Fig. 5A, white lined area, green boxes) and 180° opposite to the infarct area (i.e., the remote area, orange boxes) using Fiji software (1.51n). Tissue folds and tears were avoided as much as possible since they interfere with quantification. A threshold was set to exclude background signal of the tissue. The %area fluorescent microspheres and %area VE-cadherin were averaged over the regions of interest in the infarcted and remote area, and used to assess the extent of microvascular leakage. Slices were blinded for treatment group.

### Scanning electron microscopy

Scanning Electron Array Tomography Microscopy allows the visualisation of areas > 100 times larger than those observed in Transmission Electron Microscopy while having a comparable resolution in all three dimensions. From the mid-ventricular slices small biopsies (< 6 mm^3^) were taken from the centre of the infarct area based on CMR LGE images. In the most basal slice, an additional biopsy was taken from the posterior wall and used as an internal control. Biopsies were fixed in 2% (*v*/*v*) glutaraldehyde (Electron Microscopy Sciences (EMS)) in 0.1 M cacodylate buffer (EMS) at 4 ℃ for 24–48 h. To enhance the sample contrast for electron microscopy, an OTO based protocol was used [53, 58]. Subsequently, samples were embedded in Durcupan (See Supplemental Material for detailed information). After curing, ribbons of serial ultrathin sections (80 nm) were generated with a Leica Artos 3D Ultramicrotome and placed on Indium Tin Oxide glass coated with 5 nm carbon. Sections were examined with a Scanning Electron Microscope (Sigma300, Zeiss microscopy GmbH), using Atlas 5 software, operating at 3 kV (HDBSD, 60 µm, high current, scan speed 7, line averages 2). In 4 animals, 5 slices (on average ~ 300 µm × 300 µm, with a fixed distance between slices of ~ 1 µm) per biopsy were used for quantification, blinded for treatment group. Overview images were taken at 35 nm/pixel and the area of interstitial space, number of capillaries and extravasated erythrocytes (i.e., IMH) were quantified. Capillary density was defined as the number of visible capillaries per square millimetre. Capillaries were categorised into intact capillaries and disrupted capillaries. IMH was defined as the number of extravasated erythrocytes per square millimetre. For image processing and analysis Fiji software was used. Analyses were performed by two investigators (L.K. and A.A.) blinded for treatment group. Any discrepancy in counting was resolved via discussion. Since our fluorescent microspheres dissolve during the (heating-) protocol used for electron microscopy, we performed a pilot experiment in which gold nanoparticles (0.1 µm, 1 mL, #742031, Sigma-Aldrich) were added to the perfusion medium to assess vascular leakage. A biopsy was taken exactly in the no-reflow area as assessed by Thioflavin-S staining (Supplemental Fig. 6) and processed as described above.

### Statistics

Investigators who analysed the data were blinded with respect to the protocol, which was ensured by Castor EDC (v2022.4.1.3). To detect a clinically relevant reduction of 30% in global infarct size with *α* = 0.05, power = 0.80, standard deviation (SD) = 8, at least *n* = 6 successful experiments per treatment group are necessary for the Langendorff experiments [60]. For the in vivo experiments, at least *n* = 9 successful experiments per treatment group are necessary based on 30% reduction in infarct size, *α* = 0.05, power = 0.80, SD = 6 [64]. A dropout of 25% is taken into account. Data is presented as mean ± SD or median [interquartile range; IQR]. Figures are presented as boxplots with whiskers min to max accompanied by scatter plots for each biological replicate. When data was normally distributed based on the histograms, an independent-samples *T* test was used to test for differences between the imatinib and placebo groups. When data was not normally distributed, a Mann–Whitney *U* test was performed. A Wilcoxon signed-rank test was used to compare the infarct area to the remote area. Mixed models repeated measures was used for correlations between repeated measurements within the same animal. All results were considered statistically significant if the two-sided *p-*value was < 0.05. Statistical analyses were performed with Statistical Package for Social Sciences software (IBM SPSS Statistics 25, Chicago).

## Results

### Impact of imatinib on isolated Langendorff hearts

**Fig. 1:**
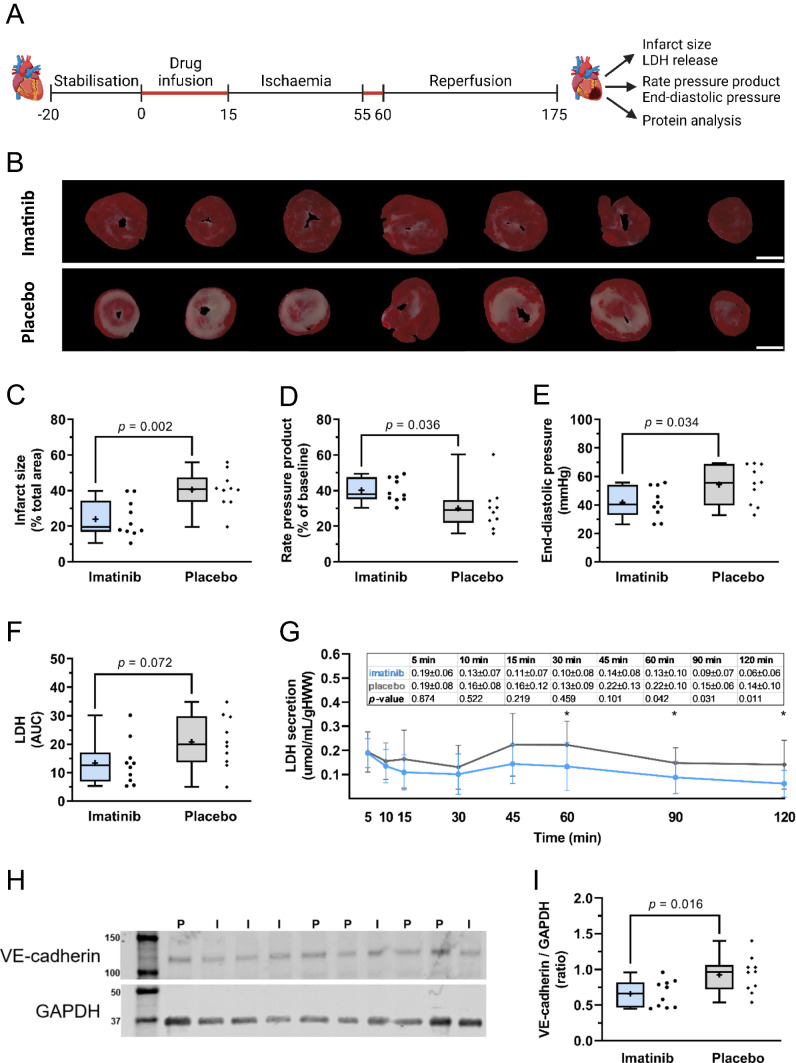
Imatinib reduces IR injury in isolated rat hearts. Isolated rat hearts were subjected to 40 min global ischaemia and 120 min reperfusion and assessed for the extent of reperfusion injury. **A** Hearts were perfused with imatinib or placebo during the first 15 min and the first 5 min of reperfusion (in red). **B** Representative images of infarct size determined with TTC staining. White scale bar represents 5 mm. **C** Imatinib reduced global infarct size, **D** improved rate pressure product recovery, and **E** decreased end-diastolic pressure at the end of reperfusion. **F** There was a trend towards reduced total LDH release in the imatinib group. **G** LDH release (umol/mL/gHWW) during reperfusion. **H** Representative Western Blots from heart apex. VE-cadherin is detected at ~ 120 kD, GAPDH at ~ 37 kD. I = imatinib, P = placebo. **I**, Relative densitometric graph after normalisation to GAPDH. Every ● represents one rat (**C**, **D**, **E**, **F**; blue, imatinib *n* = 10; grey, placebo; *n* = 10). The plus sign represents the mean. Data is presented as median with IQR, whiskers min to max (**C**, **D**, **E**, **F**, **I**) or mean ± SD (**G**), *p < 0.05, **p < 0.01, and assessed by independent-samples *T* test (**C**, **D**, **E**, **F**, **I**) or mixed models repeated measures (**G**). *AUC* area under the curve during reperfusion, *gHWW* gram heart wet weight

To investigate the direct effects of imatinib on isolated rat hearts using the Langendorff system, a total of 27 animals were operated, of which three rats were excluded from analysis due to a heart isolation period ≥ four minutes. Of the remaining 24 rats, 12 were randomised to the imatinib group and 12 to the placebo group. One rat was excluded from analysis because of technical issues during the first minutes of baseline recordings (imatinib: *n* = 1), one because of rupture of the left ventricular balloon during the experiment (placebo: *n* = 1), one because of precipitation of the Krebs–Henseleit buffer during the experiment (imatinib: *n* = 1), and the last one because of a baseline coronary flow ≤ 10 mL/min (placebo: *n* = 1). The isolated rat hearts were exposed to imatinib or placebo as described in Fig. 1A.

Groups were not different at baseline (Supplemental Table 2). Imatinib administered prior to ischaemia resulted in a significant lower vascular resistance and significant higher coronary flow compared to placebo (Supplemental Fig. 1A, B, respectively).

### Ischaemia–reperfusion injury

Imatinib treatment significantly reduced global infarct size compared to placebo (24.1 ± 10.3 vs 40.5 ± 10.3% of total heart area, *p* = 0.002, Fig. 1B, C). Total LDH secretion did not significantly differ between groups (*p* = 0.072, Fig. 1F). Subsequent LDH analysis for each separate time point showed significant lower LDH peak secretion at 60, 90 and 120 min reperfusion with imatinib treatment compared to placebo (Fig. 1G).

Furthermore, imatinib improved rate pressure product recovery (40.2 ± 6.6 vs 30.1 ± 12.4% of baseline, *p* = 0.036) and reduced end-diastolic pressure (41.7 ± 10.7 vs 54.3 ± 13.6 mmHg, *p* = 0.034) compared to placebo (Fig. 1D, E, respectively), indicating an improved cardiac function with imatinib treatment. In the apex of the heart, imatinib showed significantly less protein expression of VE-cadherin, normalised to the housekeeping gene GAPDH, compared to placebo (0.66 ± 0.19 vs 0.93 ± 0.25, *p* = 0.016, Fig. 1H, I, Supplemental Fig. 2).

### Impact of imatinib on in vivo hearts

**Fig. 2:**
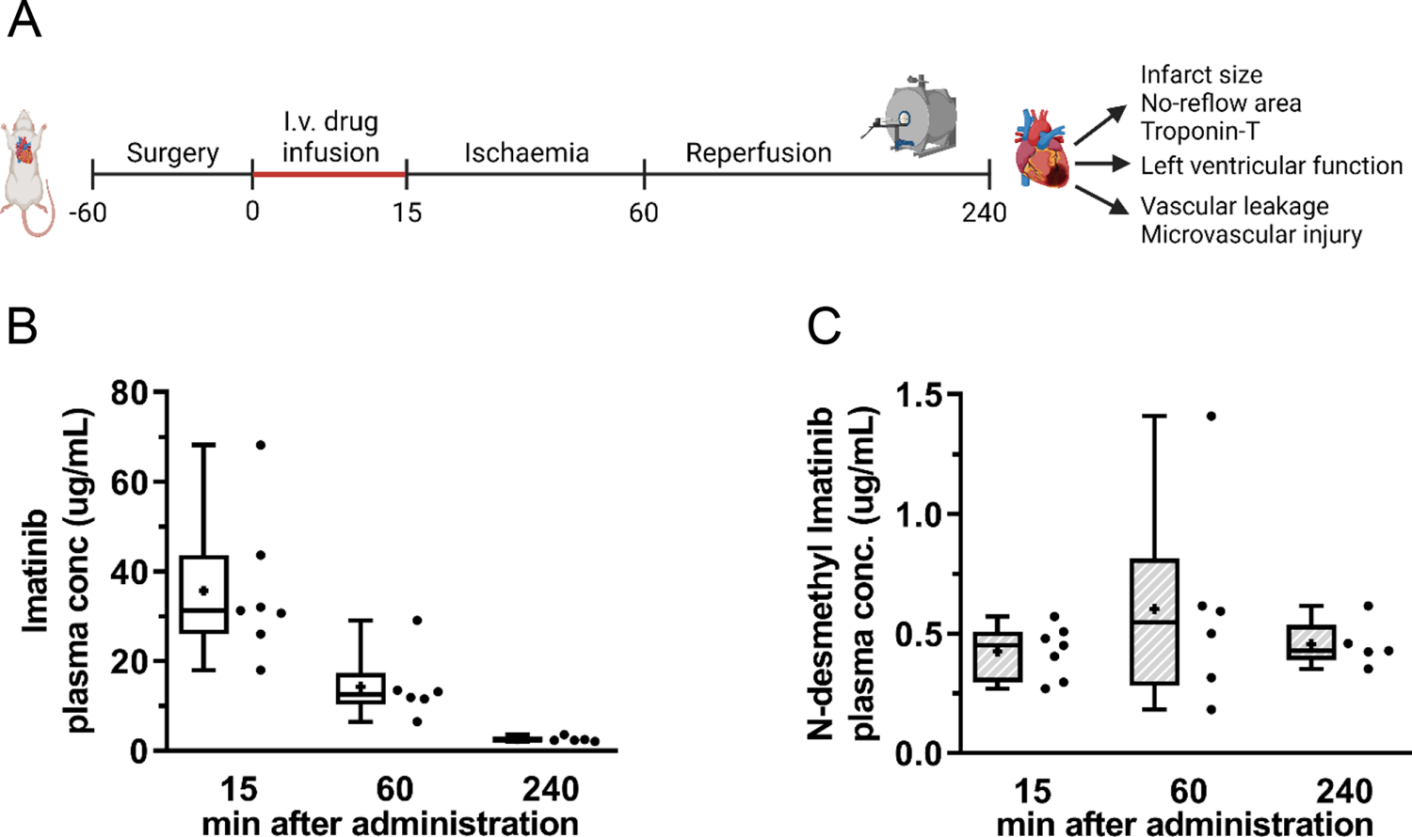
Imatinib is present in blood plasma during ischaemia and reperfusion. Rats were subjected to 45 min left anterior descending coronary artery ligation and 180 min reperfusion, and assessed for the extent of reperfusion injury. **A** Intravenous administration of one bolus of 30 mg/kg imatinib or placebo 15 min prior to ischaemia. **B** Blood plasma concentration of imatinib and **C** its main metabolite N-desmethyl imatinib were determined at 15 (end of baseline), 60 (end of ischaemia) and 240 (end of reperfusion) minutes after imatinib administration. Every ● represents one rat with *n* = 7 (15 min), *n* = 6 (60 min) and *n* = 5 (240 min). The plus sign represents the mean. Data is presented as median with IQR, whiskers min to max

Moving towards a more translational approach, the effect of imatinib on IR injury in an in vivo rat model with LAD ligation was investigated (Fig. 2A). For Cohort A: In total, 23 rats were operated, of which 11 were randomised to the imatinib group and 12 to the placebo group. One rat died during the experiment (placebo: *n* = 1). In one rat, stable ST-elevation on ECG during LAD ligation could not be maintained (placebo: *n* = 1). Due to (a small) aortic dissection during in-situ cannulation, three rats could not be used for Evans-blue/Thioflavin-S/TTC staining and assessment (imatinib: *n* = 2, placebo *n* = 1). For Cohort B: In total, 25 rats were operated, of which 13 were randomised to the placebo group and 12 to the imatinib group. Four rats died during the experiment (imatinib: *n* = 1, placebo: *n* = 3) and one rat showed no signs of ischaemia on ECG during LAD ligation (imatinib: *n* = 1). One rat could not be imaged due to CMR technical issues (placebo: *n* = 1) and one LGE CMR scan was excluded due to poor image quality (imatinib: *n* = 1). Furthermore, five non-infarcted control rats were included as a CMR reference group. Animal and procedural characteristics are summarised in Supplemental Tables 3A, B.

In the imatinib group, one bolus of imatinib (30 mg/kg bodyweight) was administered intravenously 15–20 min prior to ischaemia which led to a blood plasma concentration of 2.6 ± 0.6 µg/mL, corresponding to 5.2 ± 1.2 µM, during reperfusion (Fig. 2A). Imatinib was actively converted to N-desmethyl imatinib (Fig. 2B) and its mean concentration remained constant during the ischaemic and reperfusion phase.

### Ischaemia–reperfusion injury

Cardiac troponin-T (*p* = 0.122, Fig. 4F) and LDH (1624 [928–3115] vs 2832 [1002–4577] U/L, *p* = 0.202) were not significantly different between the imatinib and placebo groups.

### Cohort A

**Fig. 3:**
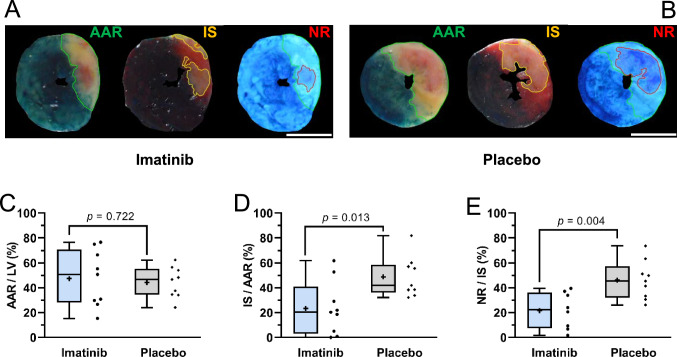
Cohort A: Imatinib reduces IR injury in vivo as assessed by triple staining with Evans-blue/Thioflavin-S/TTC. Evans-blue delineates the area at risk (AAR, green line), TTC delineates the infarct area (IS, yellow line), and Thioflavin-S delineates the area of no-reflow (NR, red line). White scale bar represents 5 mm. Representative images of a rat of **A**, imatinib group and **B**, placebo group. **C**, Area at risk as percentage of left ventricle is comparable between groups. Imatinib significantly reduced **D**, infarct size as percentage of area at risk, and **E**, no-reflow area as percentage of infarct size, compared to placebo. Every ● represents one rat (blue, imatinib *n* = 9; grey, placebo; *n* = 9). The plus sign represents the mean. Data is presented as median with IQR, whiskers min to max, and assessed by independent-samples *T* test (**C**, **D**, **E**). *LV* left ventricle

During the experiment, there were no significant differences between groups observed in heart rate, body temperature, tidal volume and oxygen saturation (Supplemental Fig. 3). Using triple staining with Evans-blue/Thioflavin-S/TTC (Fig. 3A, B), imatinib reduced infarct size (23.4 ± 21.8 vs 48.8 ± 16.2% of AAR, *p* = 0.013) and area of no-reflow (21.6 ± 14.6 vs 46.1 ± 15.5% of IS, *p* = 0.004) (Fig. 3D, E, respectively).

### Cohort B

First, to validate our in vivo CMR rat model of AMI, cardiac function of the placebo group was compared to control rats (Table 1). The placebo group showed significantly lower LVEDV and GLS, and higher LV mass despite comparable age and body weight (Supplemental Table 3B) compared to controls. Furthermore, the placebo group showed a trend for lower SV, CO and global circumferential strain compared to controls, indicating worse cardiac function.

**Table 1:** CMR characteristics for control rats, imatinib and placebo treated rats

	Control (*n* = 5)	Imatinib (*n* = 10*)	Placebo (*n* = 9)	*p*-value Control vs Placebo	*p*-value Imatinib vs Placebo
Heart rate (bpm)	370.6 ± 36.2	372.6 ± 41.6	357.7 ± 35.5	0.53	0.41
LVEDV (mL)	0.32 ± 0.01	0.30 ± 0.04	0.28 ± 0.04	***0.040***	0.48
LVESV (mL)	0.13 ± 0.01	0.12 ± 0.03	0.13 ± 0.03	0.88	0.55
SV (mL)	0.20 ± 0.02	0.18 ± 0.04	0.16 ± 0.04	***0.068***	0.30
CO (mL/min)	68 ± 8.4	63 ± 13.4	54 ± 13.3	***0.064***	0.18
LVEF (%)	60.3 ± 4.2	59.0 ± 8.4	54.0 ± 9.8	0.20	0.24
GCS (%)	− 38.0 ± 2.2	− 35.4 ± 7.5	− 31.3 ± 7.5	***0.078***	0.24
GLS (%)	− 22.0 ± 1.3	− 16.6 ± 3.1	− 15.7 ± 3.3	** < *0.001***	0.54
LV mass (mg)	378 ± 8	424 ± 67	413 ± 35	***0.017***	0.67
Infarct size (%LV)	N.a	12.6 ± 8.8	25.6 ± 9.3	N.a	***0.008***
Remote T2 values (ms)	20.2 ± 2.6	19.4 ± 4.8	19.3 ± 5.5	0.74	0.97
Infarct T2 values (ms)	N.a	37.9 ± 12.0	41.0 ± 16.0	N.a	0.65

Numbers can be written down in bold (without italics) to highlight the differences between the groups

Data is presented as mean ± SD, assessed by independent-samples *T* test

*Bpm* beats per minute, *CO* cardiac output, *EDV* end-diastolic volume, *EF* ejection fraction, *ESV* end-systolic volume, *GCS* global circumferential strain, *GLS* global longitudinal strain, *LV* left ventricle, *N.a* not applicable, *SV* stroke volume

*n = 10 for cardiac function, *n* = 9 for infarct size and T2 values

**Fig. 4:**
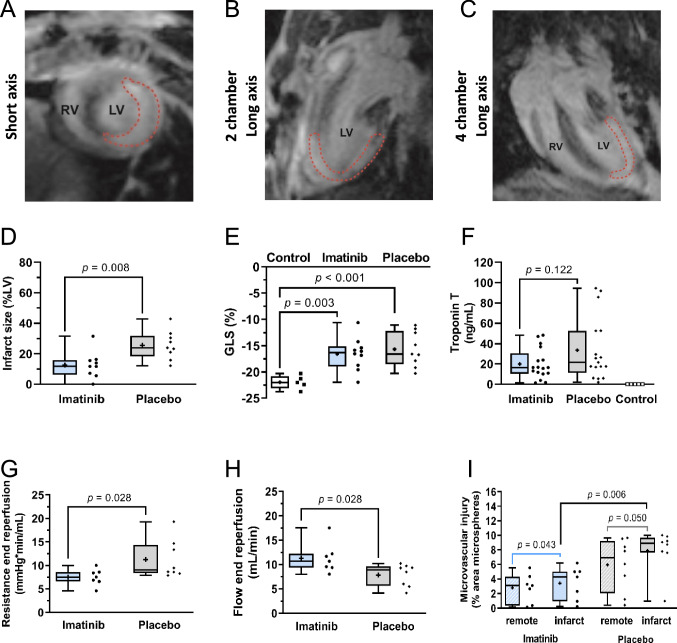
Cohort B: Imatinib reduces IR injury in vivo. Infarct size is determined with an extracellular gadolinium-based contrast agent in consecutive short axis images. Representative images of LGE CMR in **A**, short axis, **B**, 2 chamber long axis, **C**, 4 chamber long axis, in which the infarct area is marked with a red dotted line. **D**, Imatinib significantly reduced infarct size (imatinib *n* = 9, placebo *n* = 9), but **E**, did not alter global longitudinal strain (imatinib *n* = 10, placebo *n* = 9, non-infarcted control *n* = 5). **F**, Troponin T was not significantly different between imatinib and placebo (*n* = 17 in both groups; in three animals the tail vein cannula was clogged during reperfusion phase). Under controlled perfusion pressure, treatment with imatinib resulted in **G**, lower vascular resistance and **H**, higher coronary flow (imatinib *n* = 7, placebo *n* = 8). **I**, Administration of a microvascular leakage tracer showed less fluorescent microsphere extravasation in the infarct area in the imatinib group compared to the placebo group (expressed as %area of fluorescent microspheres) (imatinib *n* = 7, placebo *n* = 8). Every ● represents one rat (**D**–**I**). The plus sign represents the mean. Data is presented as median with IQR, whiskers min to max, and assessed by independent-samples *T* test (**D**, **E**, **H**), Mann–Whitney *U* test (**F**, **G**, **I**) or Wilcoxon signed-rank test to compare infarct to remote area (**I**). *GLS* global longitudinal strain, *LV* left ventricle, *RV* right ventricle

During the experiment, there were no significant differences between groups in heart rate, body temperature, tidal volume and oxygen saturation (Supplemental Fig. 4). Treatment with imatinib significantly reduced LGE CMR infarct size (Fig. 4A–C) compared to placebo (12.6 ± 8.8 vs 25.6 ± 9.3% of LV, p = 0.008 Fig. 4D), but this was not associated with changes in LVEF or GLS (Table 1 and Fig. 4E, respectively).

Since vascular resistance and coronary flow could not be determined in vivo, the hearts were isolated at the end of the CMR protocol and placed in the Langendorff system. At a constant perfusion pressure of 80 mmHg, imatinib showed a lower vascular resistance (7.5 [6.6–8.6] vs 9.0 [8.3–14.3] mmHg*min/mL, *p* = 0.028) and higher coronary flow compared to placebo (11.3 ± 3.1 vs 7.8 ± 2.3 mL/min, *p* = 0.028, Fig. 4G, H, respectively). Early microvascular leakage was visualised and quantified using fluorescent microspheres, showing less extravasation of fluorescent microspheres in the infarct area of the imatinib group compared to the placebo group (4.3 [0.95–5.0] vs 8.9 [7.6–9.7], *p* = 0.006, Fig. 4I), accompanied by higher VE-cadherin protein expression, expressed as ratio between infarcted and remote area (0.786 [0.727–0.811 vs 0.629 [0.572–0.710], *p* = 0.007, Supplemental Fig. 5B).

**Fig. 5:**
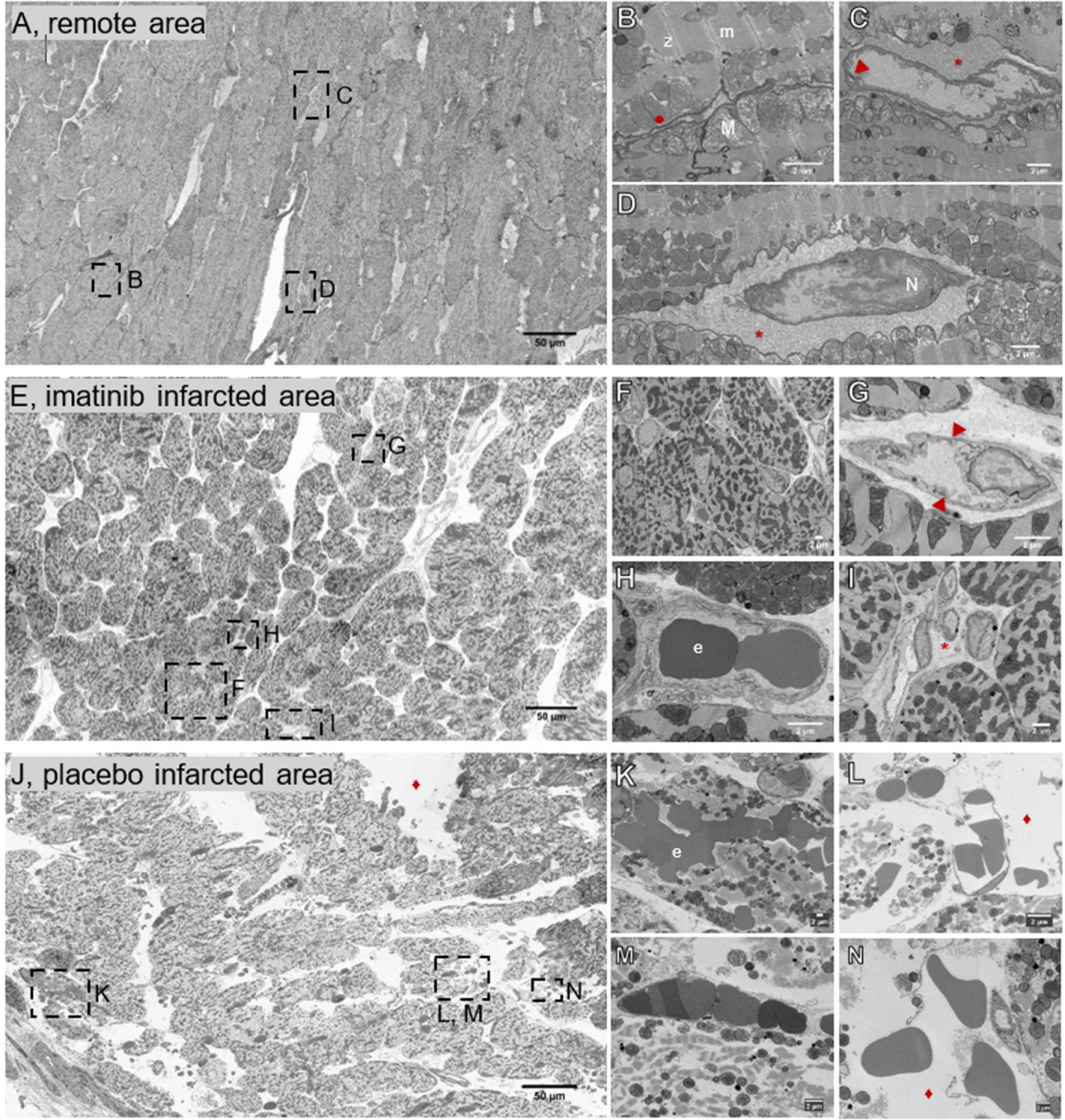
Scanning Electron Microscopy images showing the ultrastructure of the myocardium and microvasculature. **A**–**D**, remote area, **E**–**I**, imatinib treated infarct area, and **J**–**N**, placebo treated infarct area. **A**, Overview image of the remote area, with normally aligned cardiomyocytes. **B**, The cardiomyocytes showed clear gap junctions and clear Z- and M-lines of the myofibers. **C**, **D** Intact capillaries were surrounded by a dense extracellular matrix. **E**, Overview image of the imatinib treated infarct area, with large areas of normally aligned cardiomyocytes. **F**, Many intact capillaries were located close to the cardiomyocytes. **G**, Intact capillary with swollen endothelial cell, **H**, filled with erythrocytes, and **I**, surrounded by an intact extracellular matrix. **J**, Overview image of the placebo treated infarct area. The large white areas represent the interstitial space. **K**, Many erythrocytes were found in the cardiomyocytes, showing intramyocardial haemorrhage. **L**, Ruptured capillaries, **M**, obstructed capillaries, and **M**–**N**, loose myofibrils. **L**–**M** also showed a completely destructed extracellular matrix. *, extracellular matrix; ▼, endothelial cell–cell junction; e, erythrocyte; ●, gap junction; ♦, increased interstitial space; m, m-lines; M, mitochondria; N, endothelial cell nucleus; z, z-lines

**Fig. 6:**
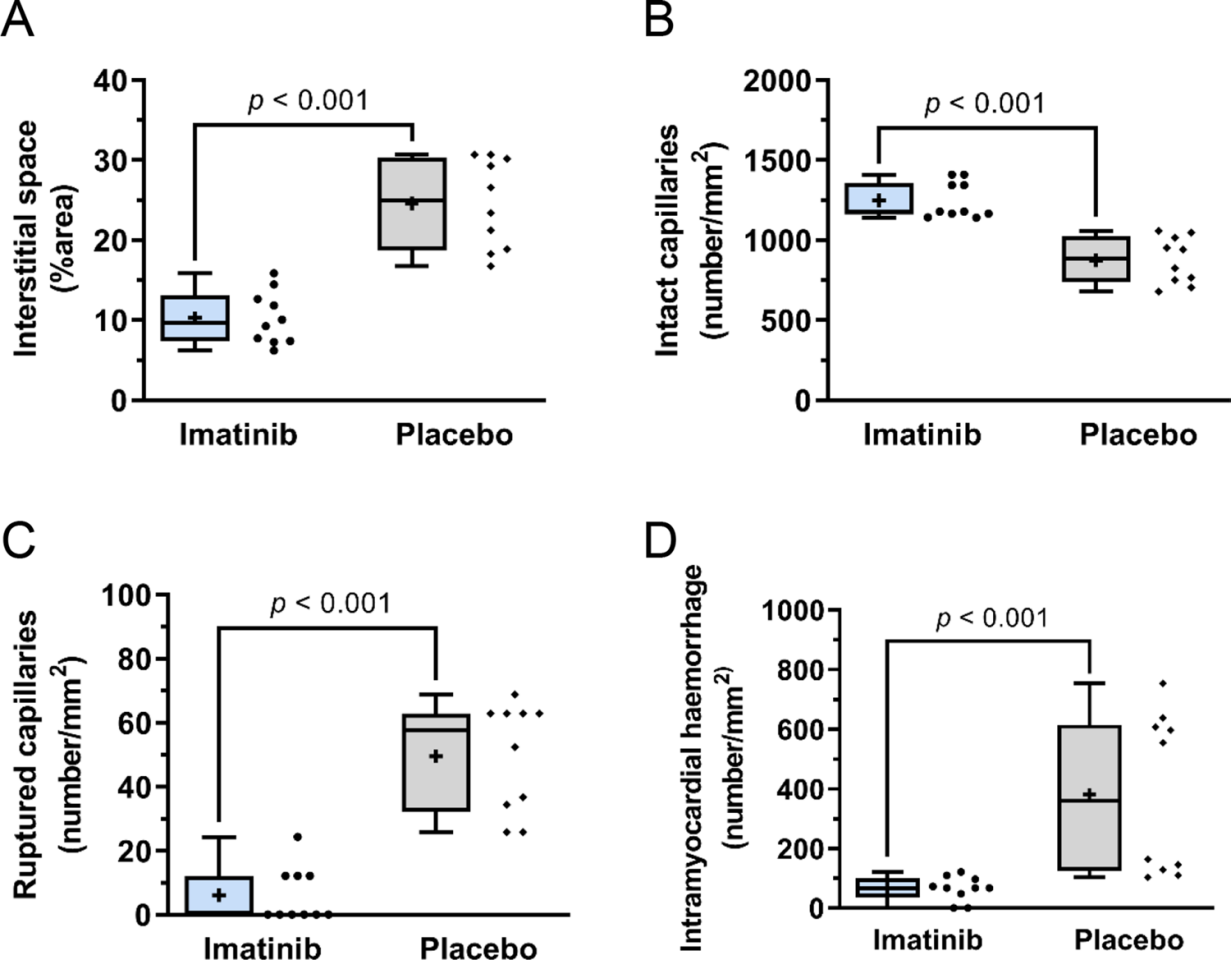
Quantification of microvascular integrity as observed with Scanning Electron Microscopy. The imatinib group showed **A**, less increased interstitial space, expressed as %area voids, **B**, more intact capillaries, **C**, less ruptured capillaries and **D**, less intramyocardial haemorrhage, expressed as number of extravasated erythrocytes, compared to the placebo group. Values are normalised to mm^2^. *N* = 2/experimental group, 5 slices/biopsy, with fixed distance of ~ 1 µm between slices. The plus sign represents the mean. Data is presented as median with IQR, whiskers min to max and assessed by independent-samples *T* test (**A**, **B**) or Mann-Whitney *U* test (**C**, **D**)

As imatinib is known to preserve cell–cell and cell–matrix interaction [3, 57], the effect of imatinib on the ultrastructure preservation of the tissue was further investigated using electron microscopy (See also Supplemental movies 1 and 2). Qualitatively, comparing the large overviews of the three groups showed clear pathological differences between the remote area (i.e., internal control area) (Fig. 5A), the imatinib infarct area (Fig. 5E) and the placebo infarct area (Fig. 5J). A general trend in increasing tissue damage was clearly visible when comparing the three samples: the most preserved sample is the remote sample, followed by the imatinib sample, and ending with the placebo sample. The most striking difference between these three samples was the increase in interstitial space, which is detected as white, empty spaces (voids). These large voids pointed at the disassociation of cardiomyocytes, suggesting interstitial oedema. Quantitatively, while the remote areas had very little interstitial space (< 5% of the total imaged area), the imatinib and placebo groups were more subject to damage (Fig. 6A).

Cardiomyocytes of imatinib treated animals mostly showed a normal area coverage. The typical coverage is demonstrated by closely aligned cardiomyocytes and prominent gap junctions (Fig. 5F)—similar to the observations for the remote sample (Fig. 5B). The imatinib subjected cardiomyocytes showed less disturbances as compared to the placebo group as assessed by the multiple mitochondria with normal size, structure and distribution, and the clearly visible Z- and M-lines of the myofibers in the imatinib and remote samples (Fig. 5B–D, F, H) in contrast to the placebo samples (Fig. 5K–M). In the placebo group, most cardiomyocytes showed signs of cell death, indicated by loose myofibrils and swollen mitochondria with loss of cristae (Fig. 5K–M).

The differences between the samples were found not only in the interstitial space and in the condition of the cardiomyocytes, but also in the integrity of the microvasculature and its surrounding extracellular matrix. In all groups (remote, imatinib and placebo), intact capillaries can be seen (Fig. 5C, D, G–I, M) with most intact capillaries located closely to the cardiomyocytes. Note, while the remote sample showed intact capillaries with clear endothelial cell–cell junctions (Fig. 5C) that are surrounded by an intact extracellular matrix (Fig. 5C, D), the imatinib samples more often showed swollen endothelial cells despite their normal appearance (Fig. 5G). The endothelial cells in the placebo group were generally thinner than those of the two other groups, most likely contributing to the fragility of the capillaries (Fig. 5M, L). Quantitatively, the imatinib group showed significantly more intact capillaries and less disrupted capillaries compared to the placebo group (Fig. 6B, C) respectively). The extracellular matrix between the capillary and the cardiomyocytes was completely disrupted in the placebo group (Fig. 5L–N) compared to the other two groups. Additionally, a substantial amount of intact capillaries in the placebo samples were clogged by erythrocytes (Fig. 5M).

Within the infarct core, there was massive extravasation of erythrocytes indicating IMH (Fig. 5K) in the placebo group; this was less in the imatinib group (Fig. 6D) and not found in the remote area in either group. Additionally, in the placebo group, erythrocytes were more frequently present within the cardiomyocytes, whereas the erythrocytes in the imatinib group were mainly limited to the interstitial space close to the ruptured capillaries (Fig. 5H). Additionally, we showed that in the no-reflow area, assessed by Thioflavin-S staining (Supplemental Fig. 6A), gold nanoparticles were translocated into the extravascular interstitial space (Supplemental Fig. 6B, C).

## Discussion

In the present study, we investigated the effects of imatinib on IR injury in the isolated rat heart and an in vivo rat model of AMI. Using the combination of an ex vivo and in vivo rat model we investigated both direct and indirect effects of imatinib on the myocardium and coronary microvasculature. Our results show that imatinib resulted in (1) smaller infarct size ex vivo and in vivo*,* (2) improved cardiac function, assessed by rate pressure product recovery and end-diastolic pressure ex vivo*,* (3) lower vascular resistance and higher coronary flow at the end of in vivo reperfusion accompanied by (4) preserved microvascular integrity. Taken together, treatment with low-dose imatinib reduced early IR injury using an ex vivo and in vivo rat model, which warrants further examining of the potential clinical impact of imatinib in the protection against IR injury*.*

Despite significant advances in the management of AMI, morbidity and mortality after AMI remain substantial [25, 27]. Hence, new cardioprotective strategies adjunct to primary PCI are still much needed. Although the majority of cardioprotective strategies have mainly aimed at reducing cardiomyocyte cell death, the protection of the microcirculation has emerged over recent years as an alternative treatment target [20]. MVI is an independent predictor of worse long-term outcome, even after adjustment for infarct size [10]. In previous studies, treatment effects on MVI were conflicting and mainly targeted endothelial cells indirectly by ischaemic conditioning, administration of vasodilators, modulation of inflammatory responses and platelet aggregation and activation [19–21, 28, 35, 44]. Increasing evidence shows that platelets not only contribute to cardiac damage, but also carry and release cardioprotective signals [28, 35]. Interventions directly targeting endothelial cells are scarce. Targeting endothelial cell adherens junctions by the use of Angiopoietin like-4 (ANGPTL4) knockout mice showed increased vascular permeability, increased extent of oedema, haemorrhage and no-reflow, and larger infarct sizes. Injecting recombinant human ANGPTL4 in a rabbit AMI model reduced the extent of haemorrhage, no-reflow and infarct size [17]. Furthermore, targeting endothelial cell adherens junctions by the Angiotensin II receptor blocker Losartan reduced vascular permeability, the extent of oedema, haemorrhage and infarct size [34]. Importantly, these studies show the relevance of additional therapeutic vascular protection after AMI, which is one of the main drivers for our experimental set-up. However, ANGPTL4 is still in the pre-clinical study phase [17], whereas losartan was only investigated in a murine model of IR injury [34].

To explore new strategies in vascular protection after AMI, we investigated tyrosine-kinase inhibitor imatinib, which is approved by the US Food and Drug Administration for patients with chronic myelogenous leukaemia and gastrointestinal tumours [4]. Imatinib targets the Arg/Abl2 kinase, which has been shown to be involved in modulating vascular leakage [2, 3, 9, 49], with an optimal protective effect on endothelial barrier function at concentrations between 5 and 10 µM [3], as also used in our study. Imatinib has been shown to be protective in a wide range of experimental animal models of vascular leakage, but has so far not been investigated in the heart with the purpose to reduce IR injury. In a sepsis model, imatinib reduced vascular leakage in the kidney and lungs [3]. In models of pulmonary IR injury, imatinib improved pulmonary function and reduced wet-to-dry ratio as a measure of vascular leakage [37, 57]. In rats that underwent cardiopulmonary bypass, imatinib reduced peripheral vascular leakage and the need for fluid resuscitation [33]. Furthermore, in ischaemic stroke models, imatinib reduced cerebrovascular leakage and infarct size [39, 56], but also haemorrhagic complications associated with the use of thrombolytic agents [56]. Interestingly, a recently published study in mice with permanent LAD ligation and subsequent heart failure showed that imatinib (30 mg/kg intraperitoneally for 3 weeks daily) alleviated long-term cardiac remodelling, inflammation and fibrosis [18], showing that low-dose imatinib can have beneficial effects in ischaemic injury without reperfusion. In line with these studies, the present study shows that imatinib is also able to reduce myocardial IR injury.

An important concern of using imatinib is its potential for cardiotoxicity [59]. Studies with in vitro cell cultures and rodent models indeed show dose-dependent cardiotoxicity of imatinib, but short-term exposure of clinically relevant concentrations (5–10 µM) in vivo were shown to be safe [8, 30, 38, 63]. Furthermore, a large, international, randomised study in patients with newly diagnosed chronic myelogenous leukaemia showed incidence rates of congestive heart failure of only 0.04% per year for patients receiving imatinib [13, 46]. Also, in a multicentre, randomised, placebo-controlled study with hospitalised patients with coronavirus disease-19, short-term treatment with imatinib (loading dose of 800 mg, followed by 400 mg/day for 10 consecutive days) for pulmonary capillary leakage showed no clinically relevant alterations in ECG parameters or cardiac biomarkers. Cardiac adverse events at 1 month did not differ between imatinib and placebo group [14]. Importantly, in our study, imatinib has been given just once and dose is substantially lower than used for oncological purposes, where imatinib is given daily for several months to years [26].

Our unique observations pertaining to the potential protective effects of imatinib on myocardial infarct size raise the question on the potential working mechanism. In previous studies, in cultured human pulmonary microvascular endothelial cells (HMVEC-L) [3, 16] and umbilical vein endothelial cells (HUVEC) [3], treatment with imatinib did not affect endothelial barrier function under basal (i.e., not-stimulated) conditions. However, under thrombin- and histamin-stimulated conditions, imatinib significantly reduced vascular permeability. Mechanistically, a well-functioning endothelial cell barrier is characterised by stable cell–cell junctions and low actomyosin tension. Treatment with imatinib reduced the formation of intercellular gaps and preserved cell–matrix interactions [3]. This is in accordance with the observations in another study with HMVEC [9], in which treatment with imatinib reduced vascular endothelial growth factor (VEGF)-, thrombin-, and histamin-induced vascular permeability. Although imatinib reduced the formation of intercellular gaps, phosphorylation status of the cell–cell junction proteins VE-cadherin and β-catenin (i.e., linked to vascular permeability [11]), was not altered. In contrast to HUVEC [3], imatinib reduced actomyosin contractility by inhibition of VEGF-induced mobilisation of intracellular calcium in HMVEC [9]. Besides its role in vascular permeability, imatinib has been shown to act as an immunomodulator in HMVEC-L treated with medium from severe acute respiratory syndrome coronavirus 2–infected airway cells [24]. Furthermore, in mouse MVEC-L, treatment with imatinib significantly increased antioxidant enzymes and significantly decreased oxidant-induced endothelial barrier dysfunction [54]. In summary, previous work in endothelial cell cultures indicate that imatinib directly targets and preserves endothelial cell barrier function, which is in line with our observations on coronary flow and vascular integrity. However, the results of the endothelial cell culture experiments should be interpreted with caution regarding myocardial IR injury. Although endothelial cell culture experiments can provide additional information on potential underlying mechanisms, the pathology of myocardial IR injury is largely determined by the high energy requirement of an intact heart. With CMR we indirectly measured vascular leakage by assessment of LV mass and T2 mapping. We showed significantly higher LV mass and infarct T2 values, indicative for increased myocardial oedema, in both experimental groups compared to our control group.

Another potential explanation for the benefits of imatinib may relate to the immediate vasodilator effects, which were already observed under resting conditions prior to ischaemia in the Langendorff experiments. These immediate vasodilator effects were also observed in a rat model of pulmonary hypertension, in which imatinib showed potent pulmonary vasodilation and reduced right ventricular pressure [1, 47]. Nonetheless, it seems unlikely that the vasodilatory properties contribute solely to the microvascular protection. Indeed, clinical trials targeting the coronary vasculature by vasodilating agents did not show a decrease in infarct size or MVI [12, 43, 62], suggesting that vasodilation alone is not sufficient to reduce IR injury.

As we previously showed, vascular leakage can be quantified by coronary infusion of fluorescent microspheres in the Langendorff system. Slow infusion of 1 mL fluorescent microspheres followed by 5 min rinsing with Krebs–Henseleit buffer showed that the majority of vascular lumina were devoid of microspheres [23]. Hence, this protocol was also used in the current study. In accordance with the previously mentioned experimental animal studies [3, 33, 37, 39, 56, 57], treatment with imatinib reduced vascular leakage compared to placebo. It is conceivable that the distribution of the fluorescent microspheres depends on the nature of the infarct: smaller infarct areas show less extravasation of the fluorescent microspheres with high intensity only in the infarct area, most likely because of better preservation of the microcirculation. In cases where IR injury is more severe, the fluorescent microspheres were extravasated in the entire tissue, suggesting that also the remote area is affected*.* Correspondingly, we observed lower expression of VE-cadherin in the infarct area compared to the remote area for both treatment groups. VE-cadherin expression was less reduced in the imatinib compared to the placebo group, which is in accordance with previously mentioned endothelial cell culture experiments. Surprisingly, in the apex of the ex vivo hearts, we found higher VE-cadherin expression with placebo. Since the placebo hearts showed larger infarcts, a possible explanation for the observed increased expression could be that the VE-cadherin promotor is activated in regions that are subjected to severe ischaemia [31, 42], but that VE-cadherin is disrupted in the infarct core. In addition, the observed reduction in vascular leakage was accompanied by a better-preserved microvascular integrity as shown by electron microscopy. Indeed, the imatinib group showed more intact capillaries surrounded by an intact extracellular matrix, less interstitial oedema and less IMH. This suggests that the observed reduction in vascular resistance and the improvement of coronary flow at the end of reperfusion may be a consequence of a better-preserved microvasculature in the imatinib group.

The protective effects of imatinib on the coronary microvasculature were accompanied by preservation of the cardiomyocytes. Both ex vivo and in vivo experiments show smaller infarcts with imatinib treatment, accompanied by less LDH release. However, troponin-T was not significantly different between imatinib and placebo, which may partly be explained by the used reagent kit. Although used in other cardioprotection studies [51], this assay detects human high sensitive troponin-T and is not validated for rats. Hence, these results need to be interpreted with caution. Importantly, the ex vivo experiments, consisting of an isolated rat heart perfused with only modified Krebs–Henseleit buffer, indicates a strong and direct effect of imatinib on cardiomyocytes and vascular endothelial cells. In addition, at this early time point (3 h of reperfusion) in the in vivo experiments, inflammatory cells are not yet present in substantial numbers [48], suggesting that the observed effects are mainly due to direct effects of imatinib on the myocardium. It is reasonable to assume that the high preservation of cardiomyocytes in the imatinib group is a result of the active role of imatinib in preserving the cardiomyocytes’ gap junctions. Furthermore, ex vivo*,* imatinib ameliorates cardiac function by improved rate pressure product recovery and lower end-diastolic pressure. Despite these beneficial effects of imatinib, LVEF did not significantly differ between control, imatinib and placebo groups during the in vivo experiments. GLS, a measure of myocardial contractility during cardiac cycle, and an early marker of damage to the cardiomyocytes [15], was significantly reduced directly after AMI compared to controls. However, GLS did not differ between the imatinib and placebo group for this specific timepoint.

### Clinical implications

Since MVI predominantly arises following acute reperfusion [23], a therapeutic window is available during the ischaemic phase (i.e., before the opening of the coronary artery). The present study shows that treatment with imatinib only 15 min before the onset of AMI resulted in less IR injury ex vivo and in vivo and preserved microvascular integrity, which facilitates translation to the clinical setting as treatment time is limited before primary PCI. Additionally, this study provides evidence that additional endothelial cell protection could be beneficial on top of timely reperfusion. Importantly, approaches that address both infarct size and MVI, as we aimed for in the present study, may attribute to improved clinical outcome in patients.

The present study has some limitations. First, as a proof-of-concept, imatinib was given prior to ischaemia and reperfusion. For better clinical extrapolation, imatinib should be given just prior to onset of reperfusion. Also, we did not include time-matched controls of isolated hearts, but rather used the non-ischaemic remote zone as control. Structural changes have also been described in the remote zone, but we were mainly interested in the differences between groups in the ischaemic zones. Furthermore, IR injury was only assessed two hours and three hours after ischaemia for ex vivo and in vivo experiments, respectively. Although IR injury increases over time [36], our experimental approach could serve as a model for early vascular leakage after AMI, even before IMH becomes visible. Additionally, this experimental approach confirms that vascular leakage is already present during the first hours of reperfusion, highlighting the potential for early treatment of IR injury by vascular protective agents. We did not perform endothelial cell culture experiments to unravel the underlying mechanism of imatinib on MVI, but assessed the expression of endothelial cell junctions and extravasation of vascular leakage tracers in the heart. Future work is necessary to confirm the observed beneficial effects of low-dose imatinib on myocardial IR injury at long-term follow-up.

## Conclusion

This study provides evidence that low-dose imatinib attenuates damage to the coronary microvasculature, reduces infarct size and global cardiac function in a rat model of myocardial IR injury. These data warrant future work to examine the potential of imatinib to reduce IR injury in patients with AMI.

### Supplementary Information

Below is the link to the electronic supplementary material.

Supplementary file1 (MP4 161370 KB)

Supplementary file2 (MP4 23439 KB)

Supplementary file3 (DOCX 4638 KB)

## Data Availability

The data underlying this article is available in the article and in its online supplementary material.
