# Supplementary material for: Imatinib attenuates reperfusion injury in a rat model of acute myocardial infarction
Source: Basic Res Cardiol. 2023 Jan 13;118(1):2. doi: 10.1007/s00395-022-00974-z (PMC9839396; doi:10.1007/s00395-022-00974-z)
Supplement: Supplementary file 3 — Supplementary file3 (DOCX 4638 KB) [file 395_2022_974_MOESM3_ESM.docx]

Basic Research in Cardiology

**Imatinib attenuates reperfusion injury in a rat model of acute myocardial infarction**

***Short title:*** ***Imatinib attenuates myocardial reperfusion injury***

Lara S.F. Konijnenberg MD, Tom T.J. Luiken MSc, Andor Veltien, Laween Uthman PhD, Carolien T.A. Kuster MSc, Laura Rodwell PhD, Guus A. de Waard MD PhD, Mariska Kea-te Lindert, Anat Akiva PhD, Dick H.J. Thijssen PhD, Robin Nijveldt MD PhD, Niels van Royen MD PhD

**Address for correspondence:**

Prof. dr. Niels van Royen

Radboud University Medical Center

Department of Cardiology

Geert Grooteplein 10, 6525 GA Nijmegen, the Netherlands

Tel: +31 24 361 4533; Fax: +31 24 363 5112; E-mail: niels.vanroyen@radboudumc.nl

**Supplemental Material**

**Detailed method Scanning Electron Microscopy**

Briefly, after rinsing, biopsies were incubated in 2% (w/v) osmium tetroxide (Structure Probe, Inc. (SPI), / 1.5% (w/v) potassium ferrocyanide (Merck) in 0.1 M cacodylate buffer with 2 mM CaCl2 (Merck) for one hour at room temperature, thereafter rinsed and incubated in 0.5% (w/v) thiocarbohydrazide solution (Sigma-Aldrich) for 30 minutes at room temperature. After rinsing, the biopsies were incubated again in 2% (w/v) osmium tetroxide for 30 minutes at room temperature, and then rinsed and incubated in 2% (w/v) aqueous uranyl acetate (Electron Microscopy Sciences (EMS)) at 4⁰C overnight. After washing, the biopsies were incubated in lead aspartate (Merck) solution (pH 5.5) for 30 minutes at 60⁰C, rinsed and dehydrated in ascending ethanol series. The dehydrated biopsies were then transferred into a mixture of acetone and Durcupan (Sigma-Aldrich) in mixtures of 3:1, 1:1, each step for 1.5 hours, followed by a 1:3 mixture for overnight. Subsequently, the samples were incubated in 100% Durcupan for 4 hours before embedding and polymerisation with fresh Durcupan.


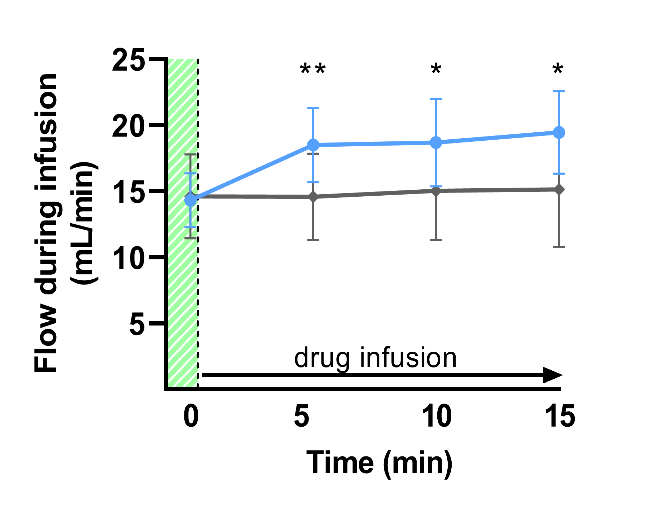


B


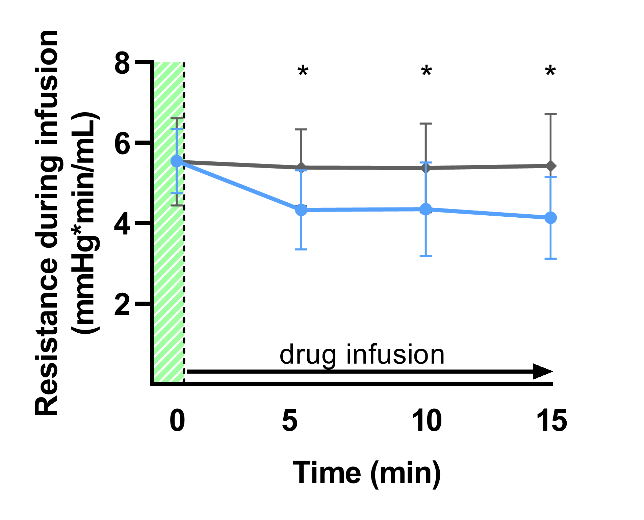


A

|  | **0 min** | **5 min** | **10 min** | **15 min** |
| --- | --- | --- | --- | --- |
| **imatinib** | 5.5±0.8 | 4.3±1.0 | 4.4±1.2 | 4.1±1.0 |
| **placebo** | 5.5±1.1 | 5.4±1.0 | 5.4±1.1 | 5.4±1.3 |
| ***p*-value** | 0.962 | 0.016 | 0.045 | 0.014 |

|  | **0 min** | **5 min** | **10 min** | **15 min** |
| --- | --- | --- | --- | --- |
| **imatinib** | 14.3±2.1 | 18.5±2.8 | 18.7±3.3 | 19.4±3.1 |
| **placebo** | 14.6±3.2 | 14.6±3.3 | 15.0±3.7 | 15.1±4.4 |
| ***p*-value** | 0.821 | 0.004 | 0.021 | 0.011 |

**Supplemental Fig 1** **Perfusion of isolated rat hearts with imatinib or placebo**

Baseline values (T=0) are depicted in the green striped area. Directly after, hearts were perfused with 10 µM imatinib or placebo for 15 minutes prior to ischaemia. **A,** Perfusion with imatinib prior to ischaemia resulted in significant lower vascular resistance and **B,** significant higher coronary flow. Blue = imatinib (n=10), grey = placebo (n=10). Data is presented as mean ± SD, * *p* < 0.05, ** *p* <0.01, assessed by mixed models repeated measures.

A


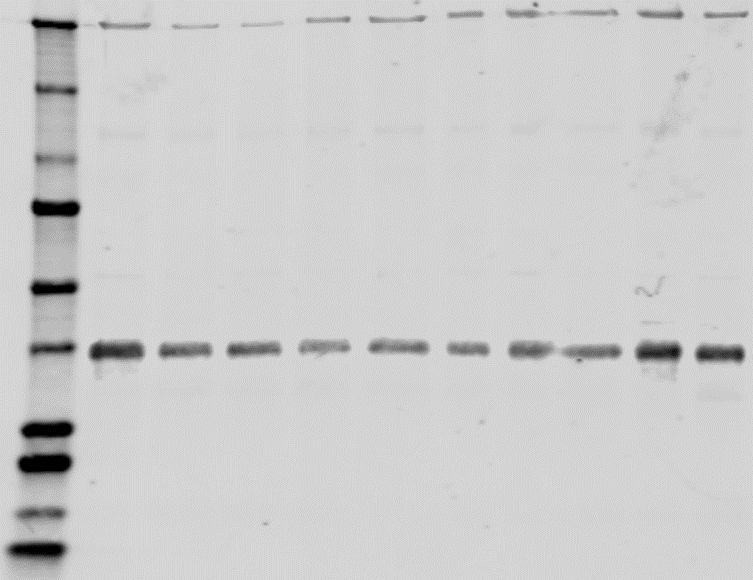

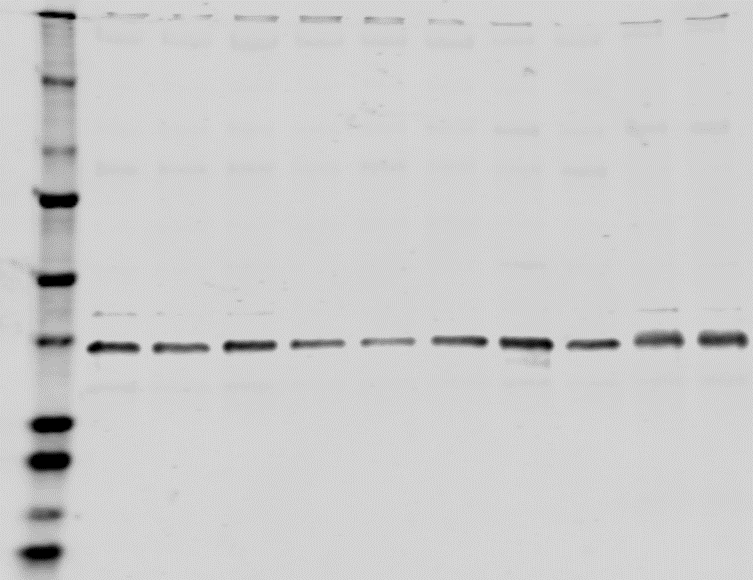


**10**

**15**

**20**

**25**

**37**

**50**

**75**

**100**

**150**

**250**

**P I I I P P I P P I**

**I P I I P I P I P P**

**GAPDH**

B


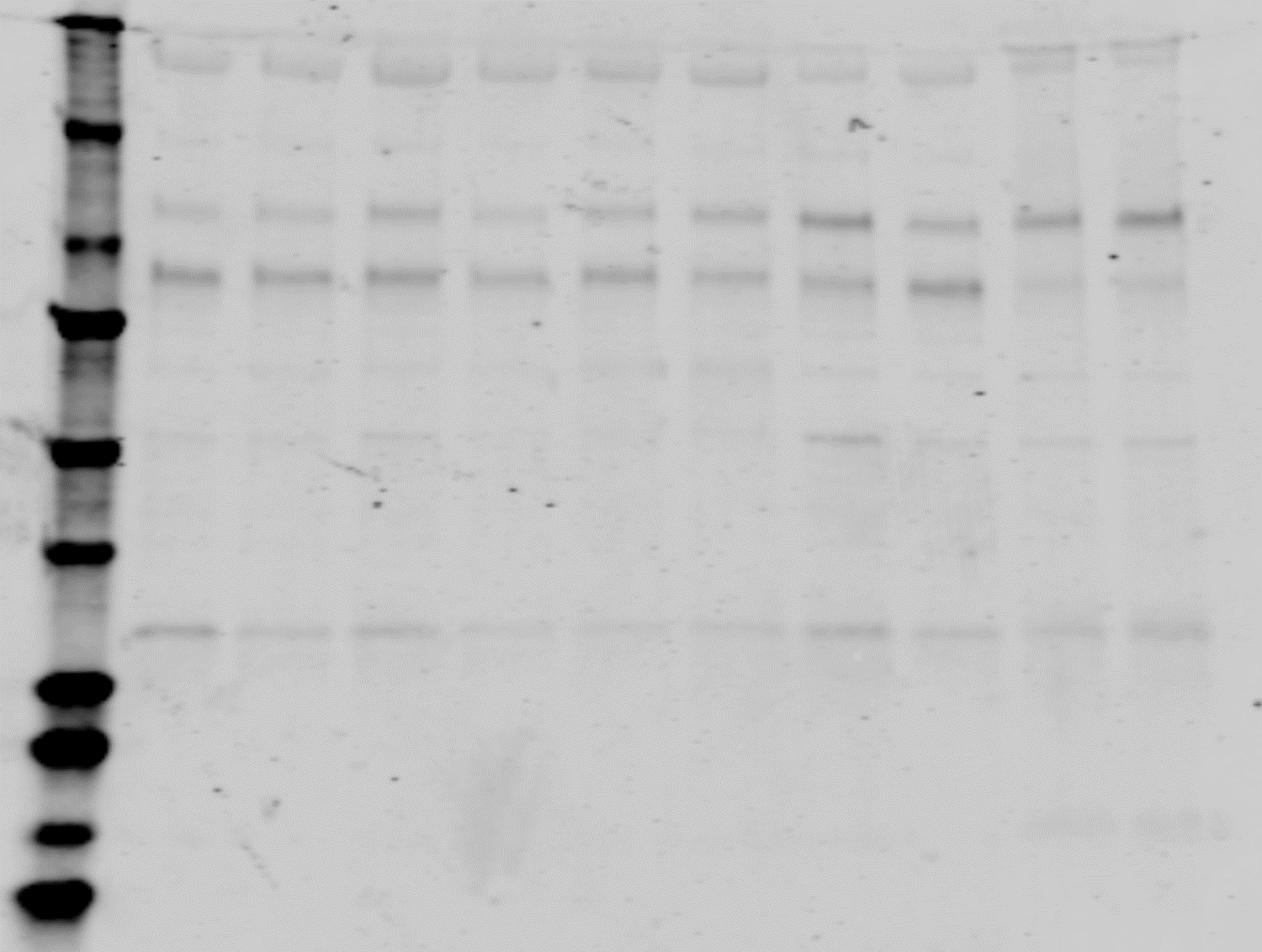

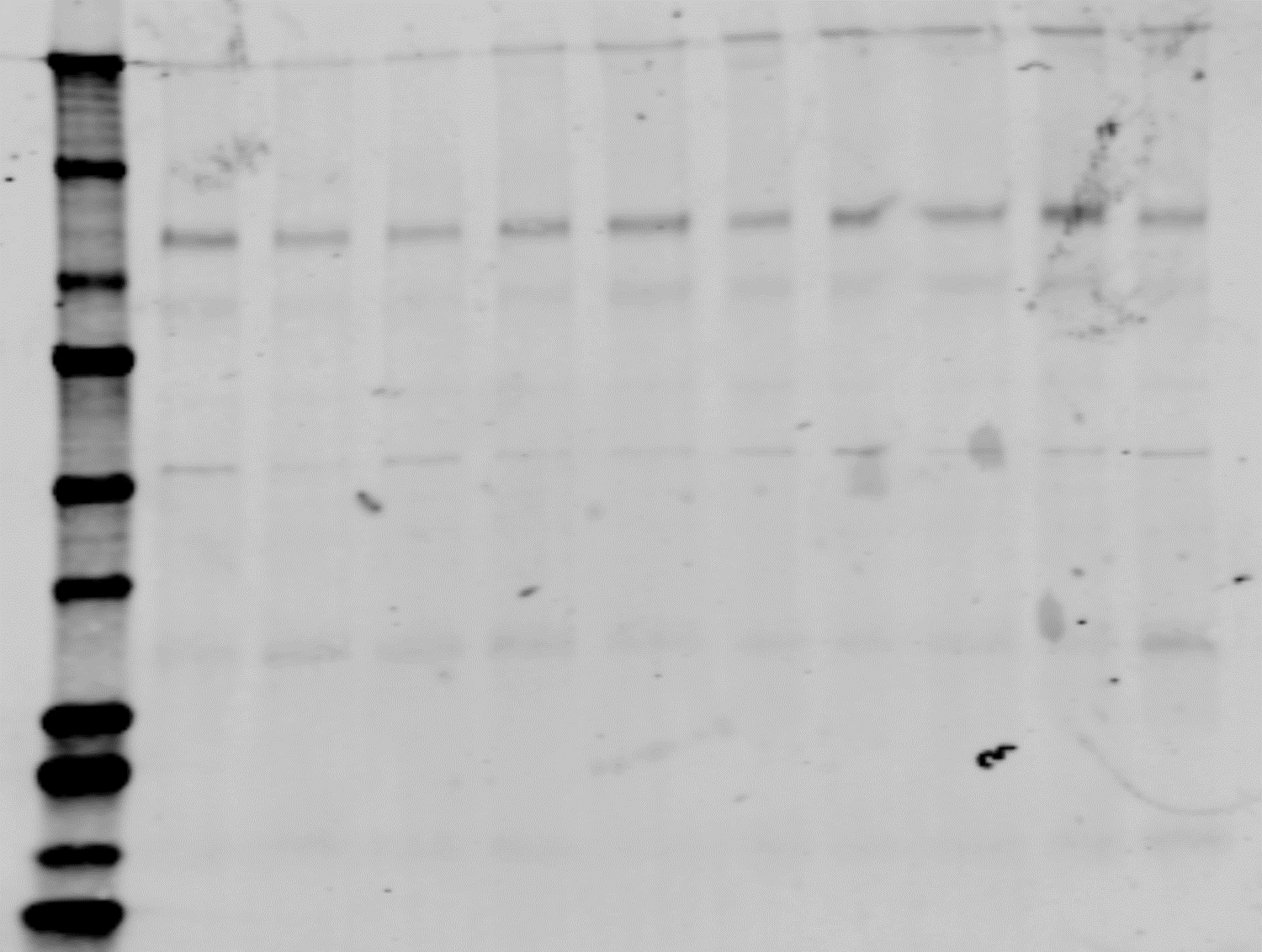


**10**

**15**

**20**

**25**

**37**

**50**

**75**

**100**

**150**

**250**

**P I I I P P I P P I**

**I P I I P I P I P P**

**VE-cadherin**

**Supplemental Fig 2 Unedited Western blots from data presented in figure 1 of the manuscript**

**A)** GAPDH for the 20 biological replicates. GAPDH was detected at ~37 kD, **B)** VE-cadherin for the 20 biological replicates. VE-cadherin was detected at ~120 kD. All samples show a non-specific band at ~250 kD. I = imatinib; P = placebo.

**
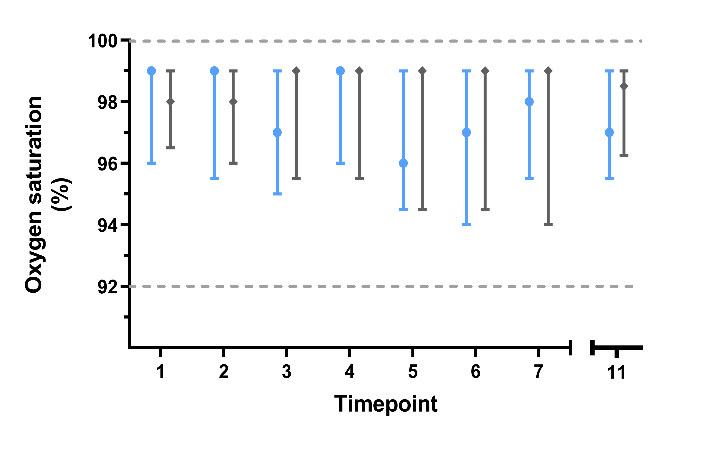
**


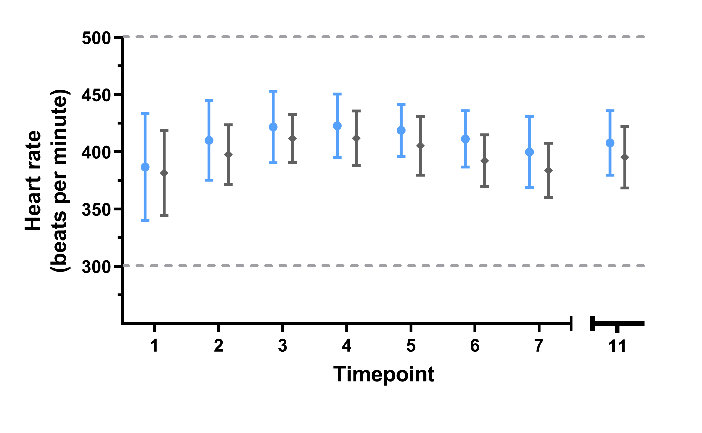

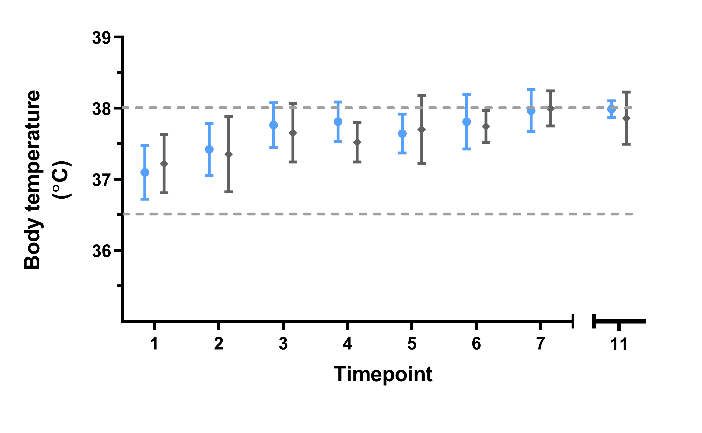

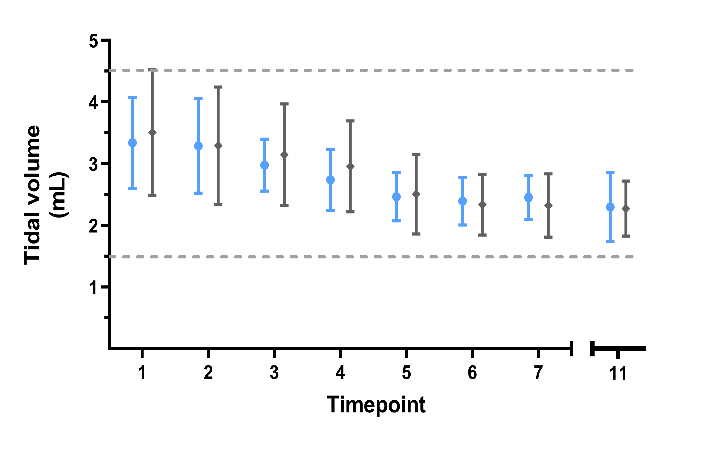


B

A

C

D

**Supplemental Fig 3 Cohort A: Haemodynamic and ventilation parameters were continuously monitored during the experiment**

Measurements of **A,** Heart rate, **B,** Body temperature, **C,** Tidal volume and **D,** Oxygen saturation. Parameters were assessed just before the mentioned time point. 1) Start thorax operation, 2) Finish thorax operation, 3) Drug infusion, 4) Start ischaemia, 5) Ischaemia t=10 min, 6) Start reperfusion, 7) Reperfusion t=10 min, 11) Heart out. Blue = imatinib (n=9), grey = placebo (n=9). Data is presented as mean ± SD (**A-C**) or median [IQR] (**D**).


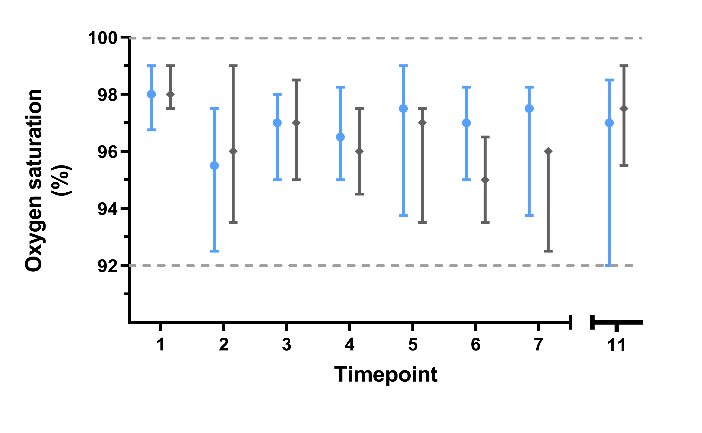


B

A

C

D


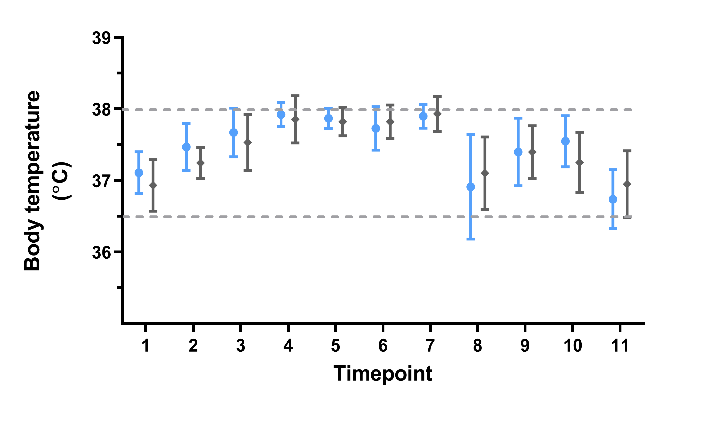

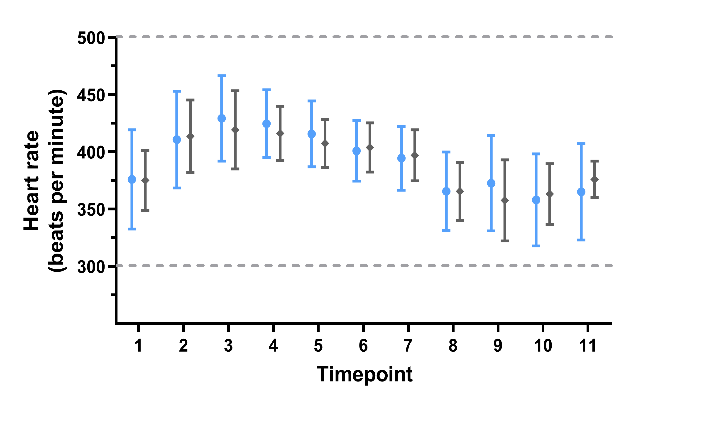

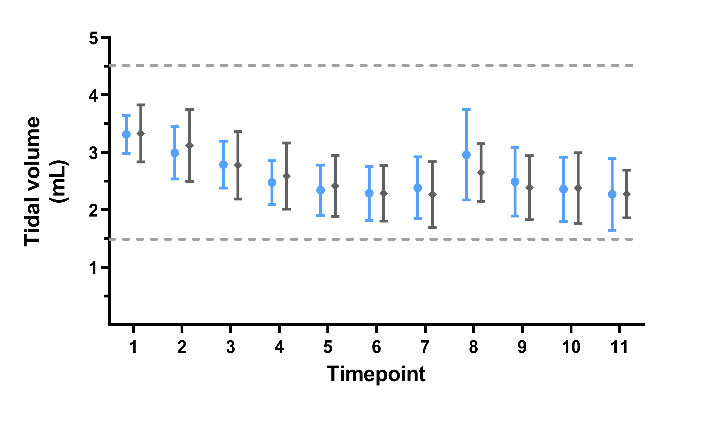


**Supplemental Fig 4 Cohort B: Haemodynamic and ventilation parameters were continuously monitored during the experiment**

Measurements of **A,** Heart rate, **B,** Body temperature, **C,** Tidal volume and **D,** Oxygen saturation. Parameters were assessed just before the mentioned time point. 1) Start thorax operation, 2) Finish thorax operation, 3) Drug infusion, 4) Start ischaemia, 5) Ischaemia t=10 min, 6) Start reperfusion, 7) Reperfusion t=10 min, 8) Start CMR, 9) Injection gadolinium-based contrast agent, 10) End MRI, 11) Heart out. Assessment of oxygen saturation during CMR was not possible. Blue = imatinib (n=10), grey = placebo (n=9). Data is presented as mean ± SD (**A-C**) or median [IQR] (**D**).

B

A

**
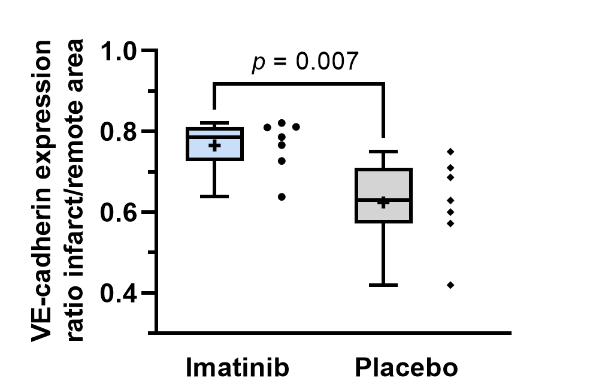
**


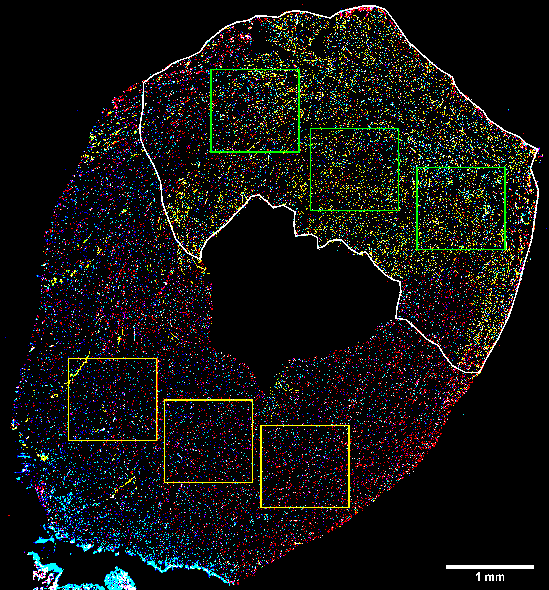

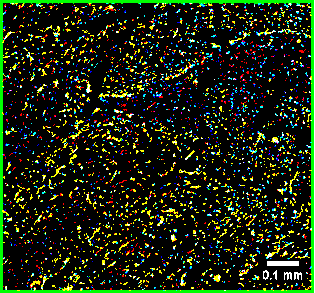

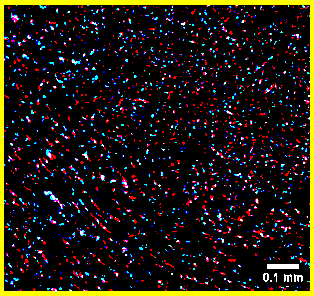


**Supplemental Fig 5 Analysis of fluorescent microspheres and VE-cadherin**

**A)** Transversal slice at the midventricular level of the rat heart. Blue = nuclei, red = VE-cadherin, yellow = fluorescent microspheres, white line = infarcted area based on CMR late gadolinium enhancement imaging, green boxes = region of interest in the infarcted area, orange boxes = region of interest in the remote area. Boxes have a fixed frame of 1*1 mm and are averaged over the respective regions. Tissue tears and folds are avoided as much as possible. **B)** VE-cadherin expression, expressed as ratio between infarcted and remote area, was less reduced in the imatinib group compared to the placebo group. Every ● represents one rat. The plus sign represents the mean. Data is presented as median with IQR, whiskers min to max, and assessed by Mann-Whitney U test.


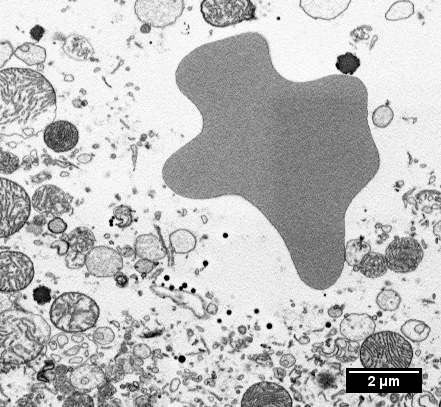


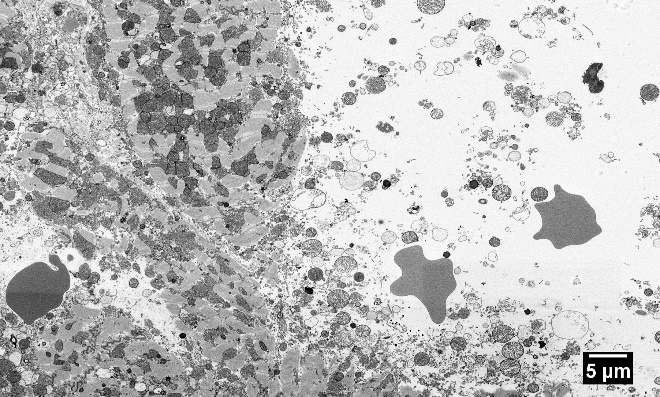

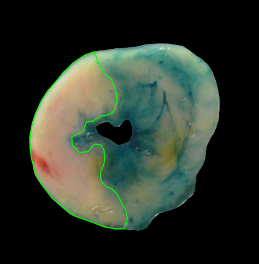

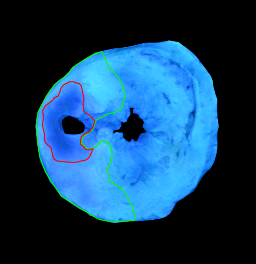

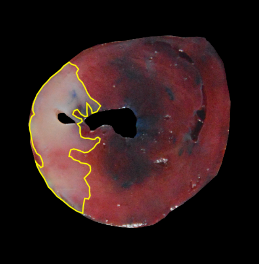


**AAR**

**IS**

**NR**

A

C

B

**Supplemental Fig 6 Extravasation of gold nanoparticles in the no-reflow area**

**A)** Evans-blue delineates the area at risk (AAR, green line), TTC delineates the infarcted area (IS, yellow line), and Thioflavin-S delineates the area of no-reflow (NR, red line). A biopsy was taken exactly in the no-reflow area (white arrow). White scale bar represents 5 mm. **B)** Overview picture of a no-reflow area with extravasation of erythrocytes and increased interstitial space. Scale bar represents 5 µm. **C)** In the no-reflow area, gold nanoparticles (100 nm, red arrows) were translocated into the extravascular interstitial space. Scale bar represents 2 µm.

**Supplemental Table 1**

| CMR scanning parameters | | | | | |
| --- | --- | --- | --- | --- | --- |
|  | **CINE**  **SAX** | **CINE 2CH/4CH** | **LGE**  **SAX** | **LGE 2CH/4CH** | **T2 map** |
| Number of slices (n) | 5 | 1 | 5 | 1 | 5 |
| Matrix | 192 x 192 | 192 x 192 | 192 x 192 | 192 x 192 | 192 x 192 |
| Field of view | 60 x 60 | 60 x 60 | 60 x 60 | 60 x 60 | 60 x 60 |
| Repetition time (ms) | 12* | 10* | 600* | 600* | 1200* |
| Echo time (ms) | 1 | 1 | 1.2 | 1.2 | 5, 10, 15 |
| Flip angle (°) | 20 | 20 | 10 | 10 | 90 |
| Inversion time (ms) | - | - | 500 | 500 | - |
| Slice thickness (mm) | 1 | 1 | 2 | 1 | 2 |
| Slice gap (mm) | 1 | - | 0 | - | 0 |
| Phases | 14 | - | 14 | - | - |
| **small variations due to ECG triggering.*  2CH, 2 chamber long axis; 4CH, 4 chamber long axis; LGE, late gadolinium enhancement; n, number; SAX, short axis. | | | | | |

**Supplemental Table 2**

| **Baseline characteristics for isolated hearts perfused with imatinib or placebo** | | |
| --- | --- | --- |
|  | **Imatinib (n=10)** | **Placebo (n=10)** |
| **Age (weeks)** | 11.0 ± 1.1 | 11.2 ± 2.4 |
| **Body weight (grams)** | 320.6 ± 38.0 | 335.9 ± 51.4 |
| **Pperf (mmHg)** | 78.0 ± 1.4 | 77.6 ± 2.1 |
| **Flow (mL/min)** | 14.3 ± 2.1 | 14.6 ± 3.2 |
| **Resistance (mmHg*min/mL)** | 5.5 ± 0.8 | 5.5 ± 1.1 |
| **Pdia (mmHg)** | 3.1 ± 3.7 | 6.1 ± 8.5 |
| **Psys (mmHg)** | 132.3 ± 19.4 | 129.3 ± 18.7 |
| **LVDP (mmHg)** | 129.2 ± 20.6 | 123.2 ± 21.9 |
| **Heart Rate (bpm)** | 268.5 ± 12.6 | 263.0 ± 18.7 |
| **RPP (LVDP * HR)** | 34716.3 ± 5899.9 | 32244.4 ± 5218.6 |
| Data is presented as mean ± SD. Bpm, beats per minute; HR, heart rate; LVDP, left ventricular developed pressure; Pdia, diastolic pressure; Pperf, perfusion pressure; Psys, systolic pressure; RPP, rate pressure product. | | |

**Supplemental Table 3A**

| **Baseline characteristics for *in vivo* experiments with Evans-blue / Thioflavin-S / TTC** | | |
| --- | --- | --- |
|  | **Imatinib (n=9)** | **Placebo (n=9)** |
| **Age (weeks)** | 9.7 ± 1.1 | 10.0 ± 0.8 |
| **Body weight (grams)** | 309.3 ± 7.5 | 311.3 ± 11.5 |
| **Heart rate (bpm)** | 386.8 ± 46.7 | 381.6 ± 37.3 |
| **Body temperature (⁰C)** | 37.1 ± 0.4 | 37.2 ± 0.4 |
| **Ischaemia (min)** | 46.9 ± 2.1 | 47.6 ± 0.9 |
| **Reperfusion (min)** | 183.6 ± 5.2 | 187.9 ± 5.2 |
| Data is presented as mean ± SD. Bpm, beats per minute | | |

**Supplemental Table 3B**

| **Baseline characteristics for *in vivo* experiments with cardiac MRI** | | | |
| --- | --- | --- | --- |
|  | **Control (n=5)** | **Imatinib (n=10)** | **Placebo (n=9)** |
| **Age (weeks)** | 10.3 ± 0.5 | 9.7 ± 0.7 | 9.9 ± 0.8 |
| **Body weight (grams)** | 307.0 ± 14.1 | 316.0 ± 8.1 | 314.8 ± 6.9 |
| **Heart rate (bpm)** | 407.8 ± 42.2 | 375.9 ± 43.4 | 375.0 ± 26.4 |
| **Body temperature (⁰C)** | 37.2 ± 0.3 | 37.1 ± 0.3 | 36.9 ± 0.4 |
| **Ischaemia (min)** | n.a. | 47.1 ± 2.6 | 46.9 ± 1.4 |
| **Reperfusion (min)** | n.a. | 187.0 [177.8 – 242.3] | 188.0 [181.5 – 193.5] |
| Data is presented as mean ± SD or median [IQR]. Bpm, beats per minute; n.a, not applicable. | | | |

**Supplemental movie 1**

The first movie shows the original electron microscopy data of the infarcted area of a placebo treated heart. This movie clearly shows ruptured capillaries, extravasation of erythrocytes, and increased interstitial space. Scale bar represents 50 um. Slice thickness is 80 nm and total volume is 320*260*6 µm.

**Supplemental movie 2**

The second movie shows the original electron microscopy data of the infarcted area of an imatinib treated heart. This movie shows predominantly intact capillaries with endothelial cell junctions. Scale bar represents 10 um. Slice thickness is 80 nm and total volume is 100*110*13 µm.
